# Gene expression analysis of *Drosophilaa Manf *mutants reveals perturbations in membrane traffic and major metabolic changes

**DOI:** 10.1186/1471-2164-13-134

**Published:** 2012-04-11

**Authors:** Mari Palgi, Dario Greco, Riitta Lindström, Petri Auvinen, Tapio I Heino

**Affiliations:** 1 Department of Biosciences, University of Helsinki, PO Box 56, Viikinkaari 5, Helsinki 00014, Finland; 2 Institute of Biotechnology, University of Helsinki, PO Box 56, Viikinkaari 9, Helsinki 00014, Finland; 3 Department of Bioscience and Nutrition, Karolinska Institutet, Stockholm, Sweden

## Abstract

**Background:**

MANF and CDNF are evolutionarily conserved neurotrophic factors that specifically support dopaminergic neurons. To date, the receptors and signalling pathways of this novel MANF/CDNF family have remained unknown. Independent studies have showed upregulation of MANF by unfolded protein response (UPR). To enlighten the role of MANF in multicellular organism development we carried out a microarray-based analysis of the transcriptional changes induced by the loss and overexpression of *Drosophila Manf*.

**Results:**

The most dramatic change of expression was observed with genes coding membrane transport proteins and genes related to metabolism. When evaluating in parallel the ultrastructural data and transcriptome changes of maternal/zygotic and only zygotic *Manf *mutants, the endoplasmic reticulum (ER) stress and membrane traffic alterations were evident. In *Drosophila Manf *mutants the expression of several genes involved in Parkinson's disease (PD) was altered as well.

**Conclusions:**

We conclude that besides a neurotrophic factor, Manf is an important cellular survival factor needed to overcome the UPR especially in tissues with high secretory function. In the absence of Manf, the expression of genes involved in membrane transport, particularly exocytosis and endosomal recycling pathway was altered. In neurodegenerative diseases, such as PD, correct protein folding and proteasome function as well as neurotransmitter synthesis and uptake are crucial for the survival of neurons. The degeneration of dopaminergic neurons is the hallmark for PD and our work provides a clue on the mechanisms by which the novel neurotrophic factor MANF protects these neurons.

## Background

Recently characterised MANF (Mesencephalic Astrocyte-derived Neurotrophic Factor) and CDNF (Cerebral Dopamine Neurotrophic Factor) form an independent family of neurotrophic factors [1]. MANF was identified from a conditioned media of cultured mesencephalic astrocytes in a search for secreted factors supporting dopamine (DA) neurons [2]. Specific loss of DA neurons is the characteristic feature of Parkinson's disease (PD). Therefore factors that maintain and support DA neurons are attractive candidates for the treatment of PD. MANF was shown to support the survival of cultured primary DA neurons but to have no effect on cultured GABAergic or serotonergic neurons [2]. Subsequently, mammalian MANF and its paralog CDNF were shown to prevent the loss of DA neurons in mouse 6-OHDA PD model [3,4]. Contrary to other vertebrate neurotrophic factors the MANF/CDNF family is evolutionarily well conserved among multicellular organisms including the fruit fly, *Drosophila melanogaster *[2,3,5]. Importantly, the protective role of MANF for DA neurons is also conserved [5]. Apparently both mammals and invertebrates share the same signalling partners as the lack of *Drosophila *Manf can be substituted by human MANF [5]. However, the interaction partners or how these proteins act at the molecular level are still elusive. It is important to understand the mechanisms of how these MANF/CDNF family proteins work at molecular level before the potential therapeutic applications.

Recent studies have shown the protective role of mammalian MANF (also known as ARMET) to be more general than restricted to the nervous system. MANF is upregulated by UPR in several mammalian cell lines [6-8] and by ischemia induced UPR in the heart and brain [8-10]. ER is the central regulator of protein folding and quality control and it has to adapt its capacity to the specific need of a particular cell type. Conditions challenging the function of the ER, like an increase of newly synthesized unfolded proteins in its lumen, lead to UPR [11]. In eukaryotes, the three canonical branches of UPR are mediated by ER membrane-associated sensor proteins. In stress-free, functional ER the intralumenal domains of these sensor proteins are bound to a chaperone BiP/GRP78 (Binding immunoglobulin protein/Glucose-regulated protein 78) and maintained inactive [12,13]. The UPR intersect with a variety of inflammatory and stress signalling pathways and networks activated by oxidative stress, all of which can influence cell metabolism. ER stress and UPR have also been implicated in the pathogenesis of several neurodegenerative diseases because of their characteristic accumulation of specific misfolded proteins [14]. Data from PD patients reveal that in DA neurons of substantia nigra the UPR is activated [15]. Recently, *Drosophila *has been used progressively to model human neurodegenerative diseases and UPR [16-18].

We have previously generated and characterized *Drosophila Manf *mutants. The zygotic null mutants (*Manf^Δ96^*) survive up to 2nd instar larvae due to the high maternal contribution. Mutants lacking both maternal and zygotic Manf (*Manf^mzΔ96^*) are late embryonic lethal and never hatch [5]. The embryonic lack of maternal *Manf *gene products and the lethality is rescued by paternal *Manf *gene expression. Both *Manf^mzΔ96 ^*and *Manf^Δ96 ^*mutants share nervous system defects, particularly dopamine neurites are altered and degenerate. Ectopic overexpression of Manf reveals no evident abnormalities in the embryonic or larval nervous system development or in the adult flies (data not shown).

Here we compare the mRNA expression profiles of *Manf^mzΔ96 ^*mutant embryos, *Manf^Δ96 ^*mutant larvae, paternally rescued maternal mutant embryos *Manf^mΔ96^/+*, and Manf ubiquitously overexpressing larvae to the wild type animals of exactly the same stages.

## Results and discussion

### The most prevalent changes in gene expression occur in *Manf *mutants that lack the maternal contribution of Manf

For microarray gene expression analysis we used two developmental stages in combination with three separate genotypes. The age of embryos and larvae were selected according to the lifespan of the *Manf *mutants. *Manf^mzΔ96 ^*mutants fail in tracheal air filling and never hatch. Mutant *Manf^mzΔ96 ^*embryos were picked during the late stage of 17 (21-22 hours after egg laying, AEL) just before hatching when the trachea of wild type embryos fill with air. Mutant *Manf^Δ96 ^*larvae with maternal contribution survive to approximately 75 hours AEL and were collected as first instars 29-50 hours AEL when maternal loads of *Manf *gene products have decreased. Three biologically independent sets of samples were used for microarray analysis. The expression profiles of all transgenic and mutant animals were compared to the wild type of the corresponding developmental stage. The numerical overview of statistically significant differences (*P *< 0.01) showed the most prominent changes in gene expression of *Manf^mzΔ96 ^*mutants (about 10% of genes differentially regulated). The smallest changes took place in the case of paternal rescue (less than 0.5% of genes differentially regulated) (Table 1). Among the differentially regulated genes, approximately half were up- or downregulated in different Manf conditions. Altogether we validated 40 genes of the microarray results. Genes were selected by several criteria such as differential expression or similar regulation in both mutants or otherwise high representation in the whole dataset. As a result, 61.5% of validated genes in different genetic conditions were independently confirmed by qPCR (Tables 2 and 3, Additional file 1: Table S1).

**Table 1:** Overview of microarray experiment

comparison between genotypes	diff. reg. probes	% all probes	**down reg**.	**% all diff. reg**.	**upreg**.	**% all diff. reg**.
***Manf*^*mz*^**^*Δ*^**^*96*^vs *wt *stage 17 embryos**	3183	7.3	1501	47.2	1682	52.8

***Manf^mz^***^*Δ*^***96*/*+ *vs *wt *stage 17 embryos**	180	0.4	53	29.4	126	70.0

***Manf^mz^**^*Δ****96***^***vs *Manf^mz^***^Δ^**^*96*^/+ stage 17 embryos**	2681	6.2	1290	48.1	1391	51.9

***Manf***^*Δ****96***^**vs *wt 29*-50 hr AEL larvae**	1734	4.0	894	51.6	840	48.4

***69B*-GAL4>*UAS-Manf*^*133 *^vs wt 29-50 hr AEL larvae**	1240	2.8	513	41.4	727	58.6

***Manf**^Δ**96**^***vs *69B*-GAL4 > UAS-*Manf^133 ^*29-50 hr AEL**	2775	6.4	1615	58.2	1160	41.8

The number of differentially regulated probes was compared. In the Agilent *Drosophila *microarray design (4 × 44 K) there was unequal number of probes targeting the particular gene, ranging from one probe to several per gene.

**Table 2:** qPCR validation of results and microarray data obtained from stage 17 embryos

		microarray		qPCR					
		***Manf*^*mz*^**^*Δ****96***^**vs *w ***		***Manf*^*mz*^**^*Δ****96***^**vs *w ***		**69B>*Manf*^*133 *^vs *w ***			

**#**	**Gene Name**	**log FC**	**P.Value**	**log2**	**T-test**	**log2**	**T-test**	**gene ID**	**Description**

**1**	*CG10420*	1.98	0.0001	5.15	9.7E-07	-3.29	1.8E-05	CG10420	Nucleotide exchange factor SIL1 precursor

**2**	*CG14879*	-4.03	7.3E-11	-3.46	0.0004			CG14879	Concanavalin A like lectin homology

**3**	*CG5810*	-1.92	1.8E-06	-3.78	1.5E-07			CG5810	leucine rich repeat

**4**	*Ddc*	1.62	4.6E-05	2.69	0.0003	-4.99	1.6E-05	CG10697	DOPA decarboxylase

**5**	*DnaJ-H*	1.10	4.1E-05	2.56	2.3E-05			CG9828	DnaJ homolog

**6**	*Hrs*	1.56	0.0001	1.38	0.05			CG2903	Hepatocyte growth factor-regulated tyrosine kinase substrate

**7**	*Hsp83*	2.46	9.7E-05	4.57	3.2E-06			CG1242	Heat shock protein 83, HSP90 homolog

**8**	*InR*	1.21	0.0007	1.87	3.4E-05			CG18402	Insulin-like receptor precursor

**9**	*Pak3*	-1.96	0.0003	-1.14	0.0003			CG14895	Pak, serine threonine kinase

**10**	*Pi3K92E*	1.11	0.0003	2.34	0.03			CG4141	phosphatidylinositol-4-phosphate 3-kinase

**11**	*pipe*	-1.53	4.2E-05	-0.25	0.0001			CG9614	heparan sulfate 2-O-sulfotransferase

**12**	*pale *	2.83	3.4E-07	4.83	1.0E-06			CG10118	TH; Tyrosine 3-hydroxylase

**13**	*punch *	1.97	4.3E-06	8.41	0.0001	0.69	0.02	CG9441	punch, GTP cyclohydrolase I

**14**	*Rala*	1.48	1.7E-07	0.63	2.1E-05			CG2849	Ras-related protein

**15**	*sulfateless *	4.21	1.8E-09	1.62	4.3E-05			CG8339	heparan sulfate glucosaminyl N-deacetylase/N-sulfotransferase

**16**	*ROP*	3.33	4.2E-10	0.82	4.3E-05			CG15811	Ras-opposite

**17**	*ubisnap *	-0.43	4.4E-05	-1.18	0.001			CG11173	ubisnap, SNAP29 homolog

**18**	*DmManf *	-1.37	0.0006	-5.39	6.3E-06	1.82	1.8E-06	CG7013	Manf, known previously as Arp-like

Only statistically significant results (P < 0.05) are presented. Results of qPCR were made comparable to microarray fold change (FC) by calculating the log2 from the relative fold changes. T-test means calculated P-value associated with Student's t-Test.

**Table 3:** qPCR validation of results and microarray data obtained from 29-50 hr larvae

		microarray		qPCR		microarray				qPCR		
		***Manf***^*Δ****96***^**vs *w ***		***Manf***^*Δ****96***^**vs *w ***		***Manf***^*Δ****96 ***^**vs 69B>*Manf^*133 *^***		**69B>*Manf^133 ^*vs *w ***		**69B>*Manf^133 ^*vs *w ***		

**#**	**Gene Name**	**log FC**	**P.Value**	**log2**	**T-test**	**log FC**	**P.Value**	**log FC**	**P,Value**	**log2**	**T-test**	**gene ID**

**1**	*DmManf*			-8.0	0.0001	-2.0	0.0002			3.2	6.0E-07	CG7013

**2**	*CG14879*	-3.2	5.5E-06	-11.5	0.0002	-2.7	4.4E-05			-3.9	1.5E-06	CG14879

**3**	*cycle*	-0.5	0.0002	-7.2	0.0001	-1.0	4.7E-09	0.5	3.2E-05	-5.0	9.0E-08	CG8727

**4**	*nol*	-1.1	0.0003	-4.3	1.7E-05	-1.9	3.9E-07	1.1	6.3E-06	0.8	0.0005	CG32077

**5**	*Pak3*	-2.8	3.1E-07	-5.8	5.2E-05	-2.7	3.8E-07					CG14895

**6**	*pipe*	-0.9	0.0003	-1.2	3.7E-06	-1.0	1.8E-05	0.9	4.1E-05			CG9614

**7**	*Rala*	-1.4	2.4E-08	-2.0	0.0001	1.4	5.7E-09	0.9	4.2E-06			CG2849

**8**	*Rep*	-2.0	0.0001	-3.3	4.6E-07							CG8432

**9**	*sip3*	-0.5	0.0003	-9.6	6.4E-06							CG1937

**10**	*Sk2*	-0.6	0.0007	-8.7	1.2E-05	-1.6	0.0003	0.6	0.0003	-12.2	4.5E-08	CG32484

**11**	*Ubc-E2H*	0.7	0.0001	1.2	9.9E-05							CG2257

**12**	*Pi3K*	-1.0	5.5E-05	-9.9	0.0003			-1.0	8.0E-05			TC208938

**13**	*Uch-L3*	-0.6	0.0003	-0.4	0.0001	-0.4	0.0004					CG3431

Only statistically significant (*P *< 0.01) results are presented. Results of qPCR results were made comparable to microarray fold change (FC) by calculating the log2 of the relative fold changes. T-test means calculated P-value associated with Student's T-test.

### Membrane transporters and metabolic genes are downregulated in *Manf^mzΔ96 ^*mutants

Development of maternal and zygotic mutant *Manf^mzΔ96 ^*proceeds until stage 16 with no differences to wild type embryos, but 21 hours AEL the cuticle and the nervous system defects become evident [5]. In comparison to wild type embryos of the same age, in *Manf^mzΔ96 ^*mutants 1191 genes were found to be downregulated. These genes were grouped into 105 functional clusters (Additional file 2), among which the most significantly enriched clusters were related to membrane transporters (25 genes) and transmembrane proteins (146 genes) (Table 4). There were several enriched clusters referring to different metabolic processes such as amine, amino acid and carboxylic acid catabolic processes (11 genes), DNA metabolic processes (26 genes), and genes related to pyrimidine metabolism (15 genes).

**Table 4:** GO clustering analysis of downregulated genes in *Manf^mzΔ96 ^*mutants

	GO term	enrichment score	gene number
**1**	membrane transporters	2.9	25

**2**	transmembrane proteins	2.3	146

**3**	amine catabolic processes	2.1	11

**4**	mitochondrion	2.0	28

**...**	DNA replication, DNA metabolic process	1.7	26

**...**	pyrimidine metabolism	1.5	15

**...**	ncRNA, rRNA metabolic process, ribosome biogenesis	1.4	23

**...**	structural constituent of chitin-based cuticle	1.4	14

**...**	Hox, DNA dependent transcription regulation	1.3	122

**...**	organophosphate, glycerolipid metabolic process	1.3	18

**...**	neurological system process	1.0	44

Clusters are represented starting from the highest enrichment score in diminishing order. Only the highest clusters are shown, for the full list of DAVID cluster analysis of downregulated genes in *Manf^mzΔ96 ^*mutants, see Additional file 2. In the case of missing order number replaced by "..." there were several clusters higher in order describing similar processes that have been listed already above.

Among the downregulated genes in *Manf^mzΔ96 ^*mutants, the gene ontology (GO) term of mitochondria-related transcripts was highly enriched (28 genes). Mitochondria are the respiratory machinery of the cell responsible for oxidation processes and participate in maintaining cellular homeostasis. The lack of Manf causes downregulation of several components in all mitochondrial compartments: the lumen as well as the inner and outer membranes.

### Stress and defence response related genes are induced in *Manf^mzΔ96 ^*mutants

In *Manf^mzΔ96 ^*mutant embryos, 1243 genes were upregulated in comparison to wild type embryos of the same stage (Additional file 3). The most prominent group of significantly enriched GO terms was immune and defense response (69 genes), followed by the groups related to proteolysis, hydrolases and peptidases (197 genes) (Table 5). The upregulated gene set was also enriched in genes related to actin cytoskeleton organization and actin filament-based process (28 genes). Moreover, genes involved in cell death (28 genes) were prominently enriched as well.

**Table 5:** GO clustering analysis of upregulated genes in *Manf^mzΔ96 ^*mutants

	GO term	enrichment score	gene number
**1**	defense response, immune response	9.4	69

**2**	endopeptidase activity, proteolysis	6.4	197

**...**	peptidase inhibitor and enzyme inhibitor activity	4.0	24

**...**	stress response, response to abiotic stimulus	3.4	31

**...**	plasma membrane part, integral to plasma membrane	3.3	59

**...**	actin cytoskeleton organization	3.1	28

**...**	extracellular region part	2.8	30

**...**	cell death	2.7	28

**...**	embryonic and epithelial morphogenesis, cell polarity	2.5	67

**...**	cell adhesion	1.7	25

**...**	membrane invagination, phagocytosis, vesicle-mediated transport	1.6	50

**...**	apical junction, cell-cell junction assembly and organisation	1.6	22

Clusters are represented starting from the highest enrichment score in diminishing order. Only the highest clusters are shown, for the full list of DAVID cluster analysis of upregulated genes in *Manf^mzΔ96 ^*mutants, see Additional file 3. In the case on missing order number replaced by "..." there were several clusters higher in order describing similar processes that have been listed already above.

### Enzymes for dopamine synthesis are upregulated despite of low dopamine levels

Extremely low dopamine levels are detected in *Manf^mzΔ96 ^*embryos [5]. Nonetheless, we observed significant upregulation of transcripts of the dopamine producing enzymes tyrosine hydroxylase (TH) and DOPA decarboxylase (Ddc) (Table 2). *Punch; *encoding a GTP cyclohydrolase, an enzyme for tetrahydrobiopterin (a cofactor for TH) synthesis was also upregulated in *Manf^mzΔ96 ^*embryos (Table 2). There could be several explanations for these alterations. Tyrosine, the essential amino acid for dopamine synthesis, is transported into the cell from hemolymph. In *Manf *mutants several amino acid membrane transporters were downregulated. The lack of substrate, tyrosine, together with low amounts of the end product, dopamine, could lead to the upregulation of the genes coding for the enzymes in dopamine synthesis pathway and their cofactors.

### Genes involved in nucleic acid metabolism and protein folding are downregulated in larval *Manf^Δ96 ^*mutants

Larval *Manf^Δ96 ^*mutants with maternally contributed *Manf *gene products never reach 3^rd ^instar stage and rarely survive up to 75 hours AEL at 25°C. Initially, *Manf^Δ96 ^*mutant larvae hatch and feed normally. Whereas wild type larvae grow rapidly, the mutant larvae remain smaller and start to wander away from food, become sluggish and stop moving, still responding to touch and usually die during the first larval molt [5]. Because of the temporal differences in survival between individual *Manf^Δ96 ^*mutant larvae from 1^st ^to 2^nd ^instar, for microarray analysis we collected larvae 29-50 hours AEL.

When comparing the expression profile of larval *Manf^Δ96 ^*mutants to the wild type larvae, almost half the number of genes (690) was significantly downregulated as compared to the rate in *Manf^mzΔ96 ^*embryos resulting in 140 functional clusters (Additional file 4). The most enriched GO terms fell into clusters related to intracellular organelle lumen and nucleic acid metabolic processes (Table 6). The cellular activities such as DNA replication, RNA processing and splicing were enriched among downregulated genes. The 5^th ^highly enriched cluster consisted of GO terms such as ER related genes (24 genes), proline and arginine metabolism (9 genes), and oxioreductases (9 genes). Mitotic cell cycle, chromosomal segregation, and mitotic spindle organization were also clustered as significantly enriched. These changes could be linked to UPR, as one of the outcomes of UPR is general and unspecific downregulation of novel protein synthesis, at the same time activating the protein synthesis for chaperones and genes enhancing the protein folding to release the unfolded protein load in ER.

**Table 6:** GO clustering analysis of downregulated genes in *Manf^Δ96 ^*larval mutants

	GO term	enrichment score	gene number
**1**	nuclear lumen, intracellular organelle lumen	3.9	50

**2**	ncRNA, rRNA metabolic process	3.7	23

**3**	chromosome, non-membrane-bounded organelle	3.7	72

**4**	DNA replication, DNA metabolic process	3.4	29

**5**	prolyl 4-hydroxylase, oxioreductase, ER part	3.4	24

**6**	RNA, mRNA metabolic process, spliceosome	2.8	45

**...**	mRNA transport, nuclear transport, nuclear export	1.6	14

**...**	chromosome condensation, DNA packaging	1.6	15

**...**	cell cycle, mitosis, chromosome segregation	1.3	59

Clusters are represented starting from the highest enrichment score in diminishing order. Only the highest clusters are shown, for the full list of DAVID cluster analysis of downregulated genes in *Manf^Δ96 ^*mutants, see Additional file 4. In the case on missing order number replaced by "..." there were several clusters higher in order describing similar processes that have been listed already above.

### Sugar metabolism, hydrolases, and ER related oxidation reduction genes are induced in *Manf^Δ96 ^*larvae

In *Manf^Δ96 ^*larval mutants, 682 genes showed upregulation in comparison to the wild type larvae. The most enriched functional clusters included GO terms like sugar metabolism and glucosidases, glycosyl hydrolases (18 genes), and hydrolases and carboxylesterases (23 genes), followed by cluster of monooxygenases, Cytochrome P450, iron, vesicular fraction, oxidation reduction and endoplasmic reticulum (49 genes) (Additional file 5). Chitin and polysaccharide metabolism was also among the highly enriched GO terms (40 genes). The 5th ranked cluster of GO terms was immune and defence response (19 genes), which was the most highly enriched cluster in *Manf^mzΔ96 ^*mutant embryos.

### Genes related to RNA metabolism, ATP binding, and DNA replication are downregulated in both *Manf *mutants

Next, we looked for functional terms among the 208 commonly downregulated genes in both *Manf *mutants (Additional file 6). There was 30% of overlap in gene sets between the *Manf^Δ96 ^*and *Manf^mzΔ96 ^*mutants. Among the downregulated overlapping genes, the enrichment of GO terms fell into RNA metabolism and ribosome biogenesis (19 genes). Around 10% of all known ATP binding genes were downregulated (28 genes) together with 14 genes of the purine and pyrimidine metabolism. Additionally, the genes coding sugar transporters and the genes involved in transmembrane transport (7 genes) highly represented among downregulated genes in *Manf^mzΔ96 ^*mutant embryos, were repressed in zygotic mutant *Manf^Δ96 ^*as well. GO terms related to DNA replication (15 genes) were also enriched among the commonly downregulated genes in both *Manf *mutants.

Altogether, the *Manf^mzΔ96 ^*mutant embryos lacking both maternal and zygotic Manf showed twice more drastic decline from wild type transcriptome than *Manf^Δ96 ^*larval mutants, whose maternal transcripts gradually diminish. Beside behavioural and growth phenotype, *Manf^Δ96 ^*larvae show degeneration of dopaminergic neurites in ventral nerve cord [5]. We found three genes downregulated in both mutants that are involved in neurite development: *Abelson tyrosine kinase *(*Abl*), *Guanine nucleotide exchange factor GEF64C *(*Gef64C*) and the transcription factor *longitudinals lacking *(*lola*).

A third of all upregulated genes (229) were induced in both mutants (Additional file 7). Immune and defense response was the most enriched functional cluster (29 genes) along with the group consisting of monooxygenases, oxidoreductases, vesicular fraction, endoplasmic reticulum, Cytochrome P450 and lipid metabolic process (21 genes). Controversially, *disabled *(*Dab*, substrate of *Abl*), was upregulated among the 10 genes involved in neuronal development *e.g*. transcription factor *Krüppel *(*Kr*), negative regulator of growth *shrub *(*shrb*), insulin receptor (*InR*), and *Drosophila *extracellular-signal-regulated kinase (ERK) *rolled *(*rl*).

### Genes related to UPR were upregulated in *Manf *mutants

Previous *in vitro *studies using tunicamycin, the inhibitor of glycosylation, to induce ER stress in mammalian cell lines have shown in UPR the upregulation of MANF [6,7]. In rat neonatal cardiomyocytes in response to UPR MANF is secreted to promote cellular survival [8]. ER stress and one of the consequences, UPR, has been mainly studied in yeast and mammalian cells. In *Drosophila*, there are several recent studies where UPR has been addressed [16,19]. Manf has been shown to be upregulated after feeding tunicamycin to adult fruit flies indicating the involvement of Manf in chemically induced UPR in *Drosophila *[20].

**Figure 1:**
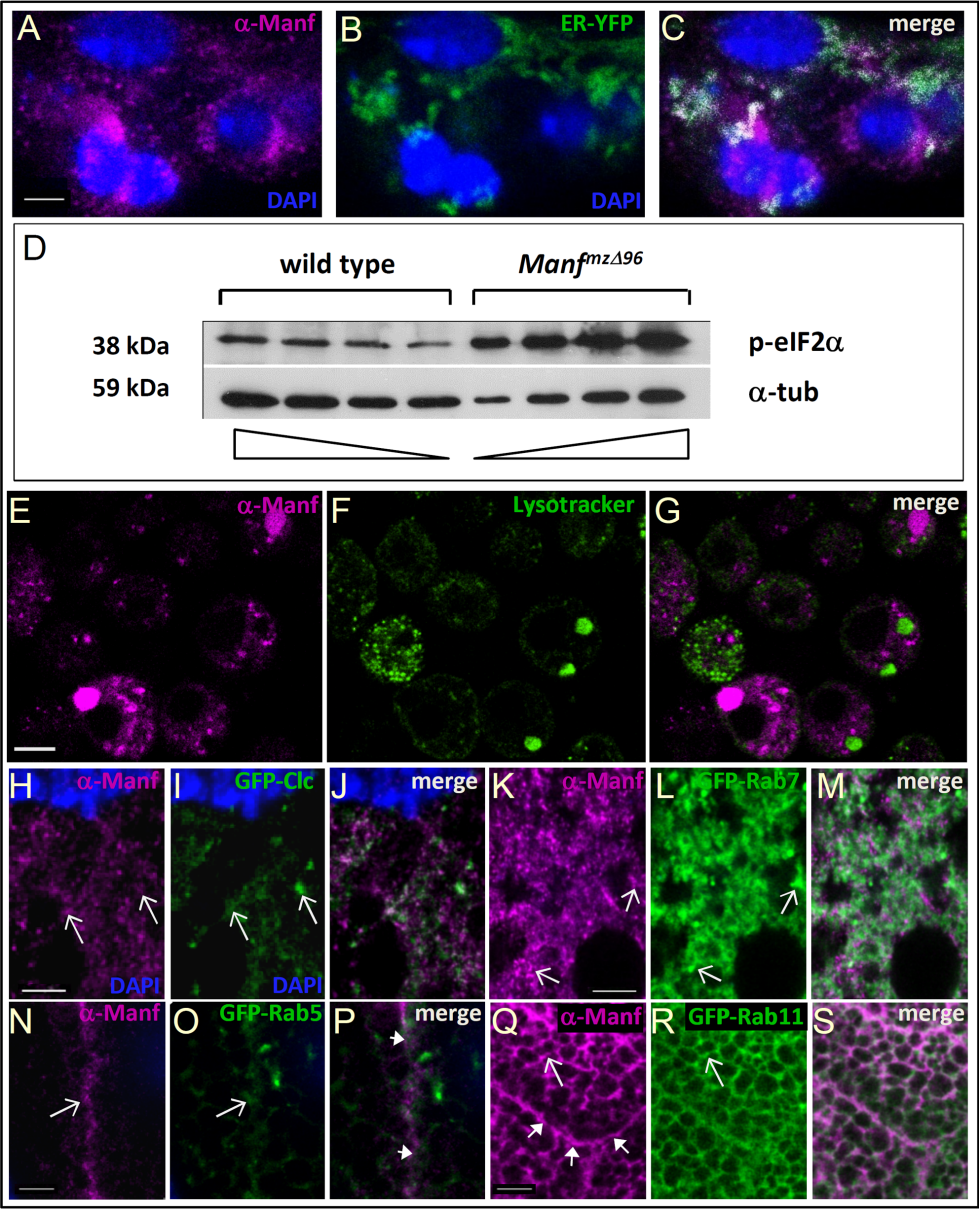
**Manf localises intracellularly partially to ER and endosomal compartment**. A-C - The confocal micrographs of 2nd instar larval garland cells stained for α-Manf (magenta) showing Manf expression around the nuclei (A) overlapping partially with ER-EYFP marker (green), DAPI (blue) was used to stain nuclei (A-C). D - Western blot analysis shows two fold increased amount of phosphorylated elF2α in *Manf^mzΔ96 ^*embryos in comparison to wild type *w^1118 ^*embryos. Decreasing amounts of samples were loaded to obtain the optimal result for quantification; the triangles represent the direction of decrease in loading. α-tubulin (α-tub) was used as a loading control. E-G - The confocal micrographs of Schneider-2 cells transfected with *Manf *cDNA construct and stained with Lysotracker (green) and α-DmManf show almost no colocalisation (less than 0.3%). H-M - The confocal micrographs of the wild type 3rd instar larval fat body expressing GFP-tagged UAS-constructs (green) driven by fat body specific *ppl*-GAL4 and stained for α-DmManf (magenta); nuclear stain DAPI (blue) was used. In H-J Manf localises close to clathrin coated vesicles marker GFP-clathrin light chain (Clc). In K-M Manf shows partial colocalisation with late endosomal compartment marker Rab7. N-S - In the salivary gland cells of 3rd instar larvae Manf (magenta) localises close to the basal cell borders and colocalises partially with early endosomal marker Rab5 (green) (N-P) and the recycling endosomal pathway marker Rab11 (green) (Q-S). Close arrows mark the cell borders and the open arrows mark the areas of colocalisation; all images consist of single laser confocal section. Scale bars: in A-C 2 μm, 4 μm in H-J, 5 μm in E-G and K-S.

To find out the intracellular localisation of Manf in *Drosophila*, we used larval 2^nd ^instar garland cells. Garland cells are nephrocytes with high rate of endocytosis and express several neuronal and exocytosis markers *e.g. prospero *(*pros*, mammalian *Prox-1 *homolog), SNARE binding protein *Ras opposite *(*Rop*) facilitating neurotransmitter secretion, and *Syntaxin 1A *(*Syx1A*, a t-SNARE) [21,22]. These cells have the most abundant expression of Manf starting from embryogenesis [5]. In the garland cells, Manf was localised around the nucleus, partially overlapping with ER-targeted marker (Figure 1A-C).

**Figure 2:**
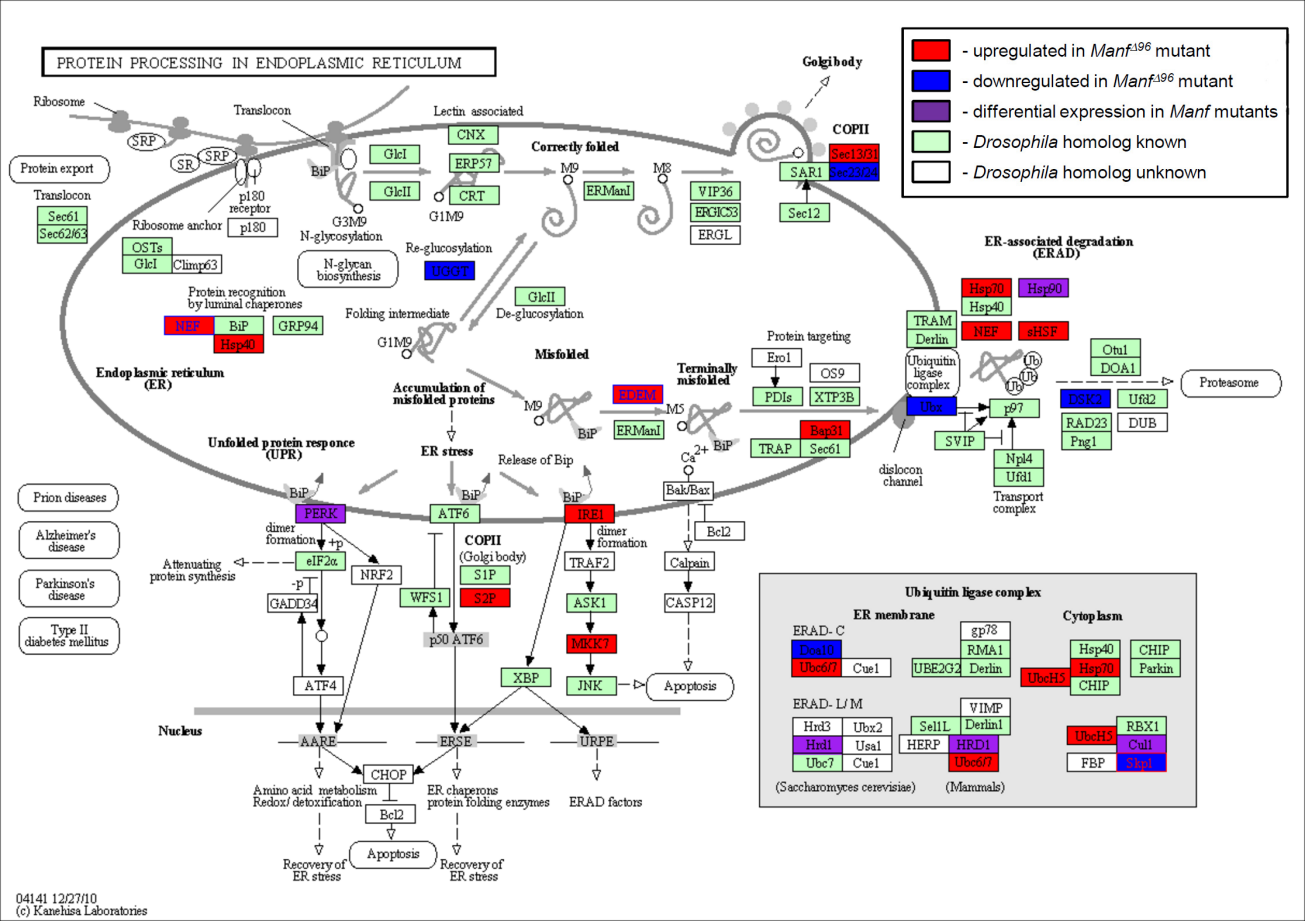
**Protein processing in endoplasmic reticulum is altered in *Manf *mutants**. An online coloured KEGG pathway scheme showing altered gene expression in red (upregulation), blue (downregulation), or purple (altered gene expression, differences between the two mutants) boxes. The unaltered known *Drosophila *homologues to identified components from other organisms are presented in green-filled boxes. The complete list of altered genes is summarised in Table 7. Notice the upregulation of genes encoding BiP/GRP78 chaperone binding proteins and components in ER leading to terminally misfolded protein degradation pathway. Out of the three branches of ER stress, IRE1 pathway leading to cell death shows upregulation in both mutants as PERK pathway is upregulated in maternal and zygotic *Manf *mutants.

**Figure 3:**
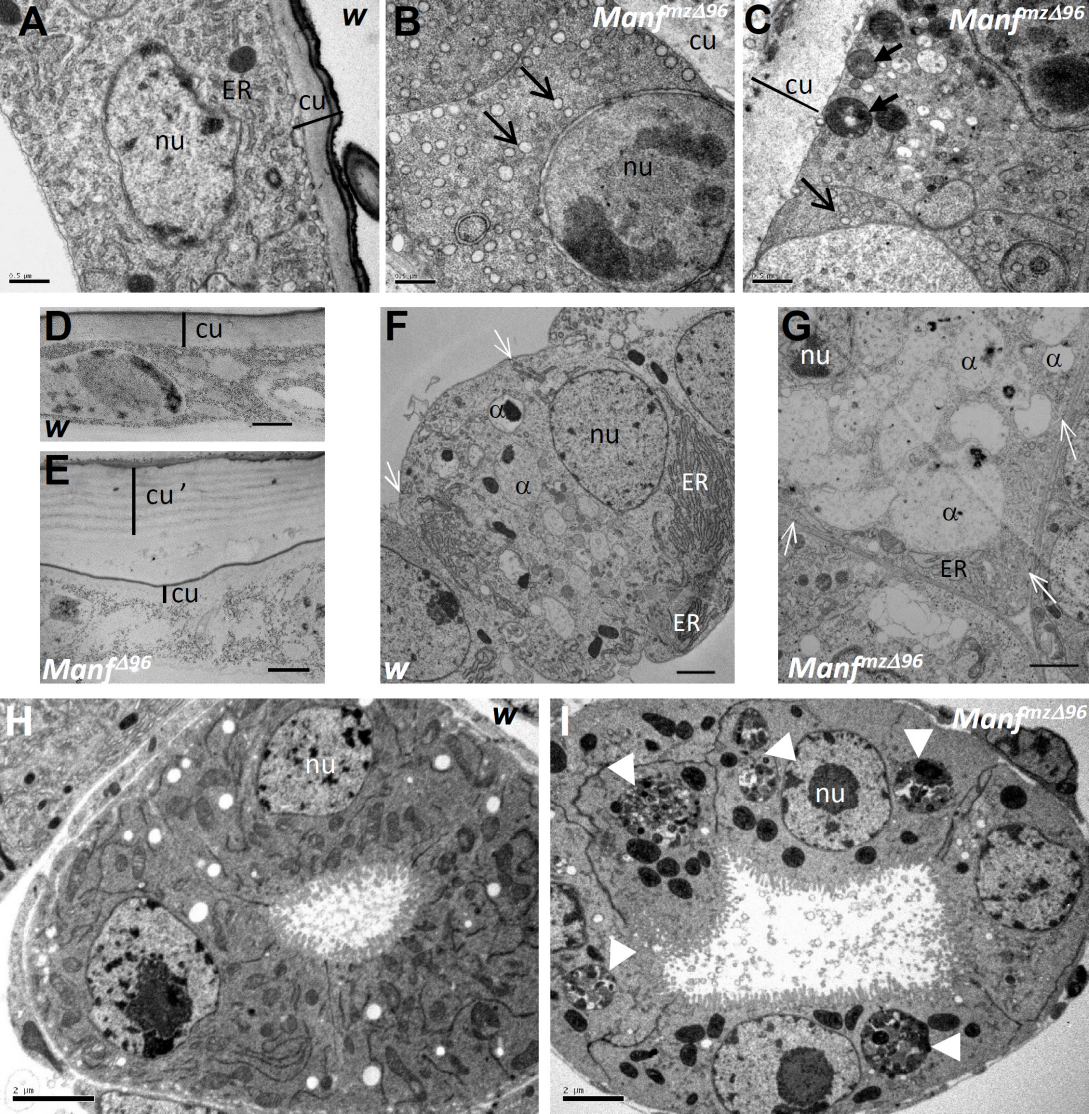
***Manf *mutants show severe defects in vesicular traffic in the cells with high secretion or endocytosis rate**. A-C - In the epidermal cells of stage 17 *Manf^mzΔ96 ^*mutant embryos compared to wild type (*w*), ER is rounded and swollen (open arrow), there are multiple vesicles stuck close to plasma membrane (thick arrow), and the cuticle (cu) is severely disorganized. Cuticular layers are indicated by a line. nu = nucleus D-E - High pressure freezing TEM images of 1st-2nd instar larvae show no difference in the layering of the cuticle between wild type and *Manf^Δ96 ^*mutant. The cellular membranes are weakly stained by this technique. Notice the unattached first instar cuticle in *Manf^Δ96 ^*mutant (cu' in E). F-G The comparison of wild type and *Manf^Δ96 ^*mutant garland cells shows excessive accumulation and enlargement of α-vesicles (α) and dilated ER. The labyrinth channels and slit membranes are similar between wild type and *Manf^mzΔ96 ^*mutant (white open arrows). H-I Secretory cells of gastric caeca in *Manf^mzΔ96 ^*mutant show accumulation of vesicles full of debris (white arrow heads) never found in wild type. Scale bars: in A-E 500 nm, 1 μm in F and G, 2 μm in H and I.

Next, we tested the hypothesis that the metabolic changes in *Manf *mutant could be the result of severe ER stress caused by altered expression of ER related genes. *Drosophila *genes homologous to several ER stress pathway have been identified. Out of 30 genes involved in ER and protein processing in the KEGG database, 24 have one or more homologues in fruit flies (Figure 2, Table 7). Of these UPR related *Drosophila *genes, 30% showed altered gene expression in our microarray experiment. Altogether, 29 genes involved in ER and protein processing show statistically significant expression changes. The gene *CG10420 *is an annotated gene with unknown function in *Drosophila*. Its human homologue nucleotide exchange factor *SIL1 *(*S. cerevisiae *ER chaperone homologue) is a BiP binding protein. In humans, several mutations in *SIL1 *gene disrupting the protein cause the Marinesco-Sjögren syndrome (MSS), an autosomal recessive cerebellar ataxia complicated by cataracts, developmental delay and myopathy [23]. We validated *CG10420 *by qPCR as downregulated by Manf overexpression and upregulated when Manf is abolished in *Drosophila *embryos and larvae. It has been shown by immunoprecipitation studies that mammalian MANF binds to BiP [24]. Thus it is possible that Manf and CG10420 compete in binding to BiP together with unfolded proteins. As the ectopic overexpression of Manf has no effect on fruit fly viability or nervous system development (data not shown), the diminished transcript level for *CG10420 *is not comparable to the total lack of this gene product in the MSS patients. According to our qPCR validated microarray results several other genes implicated in UPR were downregulated in larvae overexpressing Manf, such as *pancreatic eIF-2α kinase *(*PERK*)*, Heat shock protein 83 *(*Hsp83*)*, Ubiquilin *(*Ubqn*), and *septin interacting protein 3 *(*sip3*). In embryonic *Manf^mzΔ96 ^*mutants all above mentioned genes were significantly upregulated as well as considerable number of other ER chaperone genes (Table 7). Furthermore, when evaluating the ultrastructural changes in *Manf^mzΔ96 ^*mutants, we noticed that the ER was swollen and dilated in epidermal cells, indicating severe disturbances of ER structure (Figure 3A-C). In *Manf^mzΔ96 ^*mutant embryos the extent of phosphorylated eukaryotic initiation factor eIF2α was more than two fold upregulated when compared to the wild type (Figure 1D) indicating the presence of UPR in these *Manf *mutants. The phosphorylation of eIF2α by PERK is a hallmark for UPR, resulting in reversible blockage of translation and downregulation of the protein load to the ER [25]. In *Drosophila *there are two kinases, PERK and Gcn2, shown to be able to phosphorylate eIF2α [26,27]. The expression of *Gcn2 *is high only during early stages of embryogenesis [27]. Thus PERK is a potential candidate kinase behind eIF2α phosphorylation at the end of embryogenesis. Interestingly, our microarray data showed that in *Manf^mzΔ96 ^*mutants the transcription of PERK was [25] upregulated and the genes involved in different metabolic processes such as amino acid, DNA and pyrimidine metabolism were downregulated indicating overall inhibition of translation. So it is probable that the UPR PERK pathway is activated in *Manf^mzΔ96 ^*mutants. The second UPR sensor, IRE1, activates two separate downstream branches. One of the branches leads to the activation of Jun kinase and death pathway [28]. According to the microarray results, *Drosophila *Jun kinase kinase *hemipterous *showed significant upregulation in both *Manf *mutants.

**Table 7:** List of genes with altered expression according to microarray analysis related to KEGG pathway of protein processing in ER

KEGG		embryo		larvae		
name	gene	**o.ex**.	**mut**.	**o.ex**.	**mut **.	description
NEF	*CG10420*	**down**	**up**	**down**	**up**	SIL1, BiP-associated protein, mutated in MSS

Hsp90	*hsp83*		**up**	**down**	**down**	Heat shock protein 83

PERK	*pek*		**up**	**down**	**down**	PEK; pancreatic eIF-2alpha kinase, UPR sensor

Hrd1	*sip3*		**up**	**down**	**down**	septin interacting protein 3, ERAD

DSK2	*ubiqn*		**up**	**down**	**down**	ubiquilin, ubiquitin-ass./transl. elongation factor EF1B

Cul1	*lin19*		**down**	**up**	**up**	lin-19-like, ubiquitin protein ligase, cullin homology

Skp1	*skpC*		**down**	**up**	**down**	skpC, E3 ubiquitin ligase

EDEM	*Edem1*			**up**	**down**	Edem1, Glycoside hydrolase

Ubx	*p47*			**down**	**down**	p47, human NSFL1

Skp1	*skpE*		**down**		**down**	RNA polymerase II transcription elongation factor

Sec23/24	*CG1472*	**down**			**down**	COP complex II, mediator of selective export from ER

UGGT	*CG6850*				**down**	UDP-glucose-glycoprotein glucosyltransferase

DOA10	*CG1317*		**down**			E3 ubiquitin-protein ligase MARCH6

Sec13/31	*CG6773*		**up**	**up**	**up**	involved in export from ER, nuclear import, cuticle development

MKK7	*CG4353*		**up**		**up**	hemipterous, Jun kinase kinase

Hsp70	*Hspc1*				**up**	Heat shock protein cognate 1

Ubc6/7	*CG5823*				**up**	ubiquitin-conjugating enzyme E2 J2

Hsp40	*dnajh*		**up**			DnaJ homolog subfamily B member 11

NEF	*CG10973*		**up**			hsp70-interacting protein

Bap31	*CG13887*		**up**			B-cell receptor-associated protein 31

NEF	*CG2918*		**up**			hypoxia up-regulated 1

sHSF	*l(2)efl*		**up**			lethal (2) essential for life

IRE-1	*ire-1*		**up**			inositol requiring enzyme 1, Ser/Thr kinase, UPR sensor

Hsp70	*Hsp68*		**up**			Heat shock protein 68

UbcH5	*Ubce2*		**up**			Ubiquitin conjugating enzyme 2

Hsp70	*Hspc2*		**up**			Heat shock protein cognate 2

NEF	*CG7945*		**up**			BCL2-associated athanogene 2

Sec13/31	*CG8266*		**up**			COP II complex, secretory vesicle budding from ER

S2P	*S2P*		**up**			endopeptidase

Significant alterations in gene expression from wild type are shown by word code; "up" represents upregulation and "down" downregulation of gene expression. Gene name stands for the particular homologue gene name in *Drosophila*. Notice that the same KEGG identifier can lead to several different genes. p.res = paternal rescue, mut = mutant, o.ex. = overexpression.

Conclusively, the absolute lack of Manf results in severe ER stress and upregulation of many genes involved in UPR finally leading to the cell death. When maternal Manf stores are gradually decreased in *Manf^Δ96 ^*larvae, there are only few genes upregulated that are related to ER: *CG10420*, ubiquitin protein ligase *lin19*, heat shock protein cognate 1 and ubiquitin-conjugating enzyme *CG5823*. As our data come mostly from gene expression analyses, further biochemical experiments are needed to identify the exact role of Manf in UPR.

### Lack of Manf results in downregulation of several genes in exocytosis pathway

Ultrastructural study of *Manf^mzΔ96 ^*mutants revealed overload of vesicles next to the apical part of epidermal cells and reduced microvillae thought to enhance the capacity of the secretion of these cells (Figure 3C). This result, together with the severe defects observed in the cuticle secretion and organisation (Figure 3B, C), suggested a possible involvement of the genes of the exocytosis pathway. Indeed, the expression of several genes related to exocytosis and SNARE transport were altered in different Manf conditions (Table 8 Additional file 8). In *Manf *mutants, several genes implied in exocytosis and vesicle transport from Golgi complex to the plasma membrane were downregulated (*Syx1A, Syx6, SNAP29*), whereas the ER residing syntaxins - *Stx17 *and *Stx18 - *were upregulated. This supports an inhibition of secretion from Golgi complex to the plasma membrane as seen in *Manf^mzΔ96 ^*mutant epidermal cells in vesicle accumulation close to the apical area (Figure 3C).

**Table 8:** List of genes with altered expression according to microarray analysis related to KEGG pathway of exocytosis and SNARE complexes

KEGG		embryo		larvae		
name	gene	**o.ex**.	**mut**.	**o.ex**.	**mut**.	description
Bos	*CG4780*	**up**	**up**	**up**	**down**	membrin

Vti1	*koko*			**up**	**down**	kokopelli; cyclin-dependent protein kinase regulator

SNAP29	*usnp*	**up**	**down**		**down**	usnp; ubisnap

Stx6	*Syx 6*	**up**	**down**			Syntaxin 6

	*Syt7*		**down**	**down**	**down**	calcium-dependent phospholipid binding, Synaptotagmin.

Stx1-4	*Syx 1A*			**down**	**down**	Syntaxin 1A

Stx17	*Syx 17*			**down**	**up**	synaptic vesicle docking; neurotransmitter secretion

	*Synd*		**up**		**up**	neurotransmitter secretion; synaptic vesicle endocytosis

Stx13	*Syx 13*		**up**			SNAP receptor: cytokinesis after mitosis and meiosis

	*Syb*	**up**	**up**			SNAP receptor: synaptic vesicle docking in exocytosis

Syx18	*Syx 18*	**up**	**up**			Syntaxin 18

Bet1	*CG14084*	**up**	**up**			Bet1

VAMP7	*CG1599*	**up**	**up**			vesicle-associated membrane protein 7

Significant alterations in gene expression from wild type are shown by word code; "up" represents upregulation and "down" downregulation of gene expression. Gene name stands for the particular homologue gene name in *Drosophila*. Few related *Drosophila *genes associated with neurotransmission missing from KEGG pathway were added. The according coloured scheme for exocytosis and SNARE of KEGG pathway is presented in Additional File 8. p.res = paternal rescue, mut = mutant, o.ex. = overexpression.

### Expression of genes involved in cuticle development were altered in *Manf^mzΔ96 ^*mutants

We have previously shown that *Manf^mzΔ96 ^*embryos have disorganized cuticle [5]. At the end of embryogenesis from stage 16 onward, the cuticle components are secreted by epithelial cells and stored in regular layers, and subsequently the cuticular proteins are crosslinked by dopamine-derived quinones [29,30]. Among the downregulated genes in *Manf^mzΔ96 ^*embryos, there were 14 genes coding the structural components of the insect cuticle. At the same time, several other genes responsible for cuticle development were upregulated, such as the genes encoding enzymes involved in chitin synthesis, *krotzkopf verkehrt *(*kkv*, chitin synthase-1) [31,32], *knickkopf *(*knk*, N-glycosylated membrane-bound extracellular protein involved in chitin microfibril formation) [33], and *Syx1A *(responsible for cuticle component secretion). Additionally, several genes involved in epithelial development and morphogenesis were upregulated and significantly enriched among the GO terms (35 genes) (Table 5 and Additional file 3).

We used transmission electron microscopy (TEM) analysis in *Manf^mzΔ96 ^*mutants at the embryonic stage 17 to investigate the epithelial cells responsible for cuticle secretion. Indeed, these cells showed morphologically abnormal ER and accumulation of vesicles in the apical part (Figure 3A-C). It is possible that the enhanced endocytosis and disturbed exocytosis, together with misbalance in cuticular components, lead to disorganised and disrupted cuticle in *Manf^mzΔ96 ^*mutant embryos. In larval *Manf^Δ96 ^*mutant with gradually fading maternal contribution, the cuticle showed no disruption and the chitin layers were deposited and organised normally (Figure 3D, E). Instead there were problems in shedding the old cuticle and often the 1^st ^instar cuticle remained attached (Figure 3E). This implies that the maternal loading of *Manf *gene products in larval *Manf^Δ96 ^*mutants was sufficient to overcome defects in early cuticle development, secretion and layering, but insufficient to complete the first molt.

### Large vesicles filled with electron dense debris are accumulated in *Manf^mzΔ96 ^*mutant

**Figure 4:**
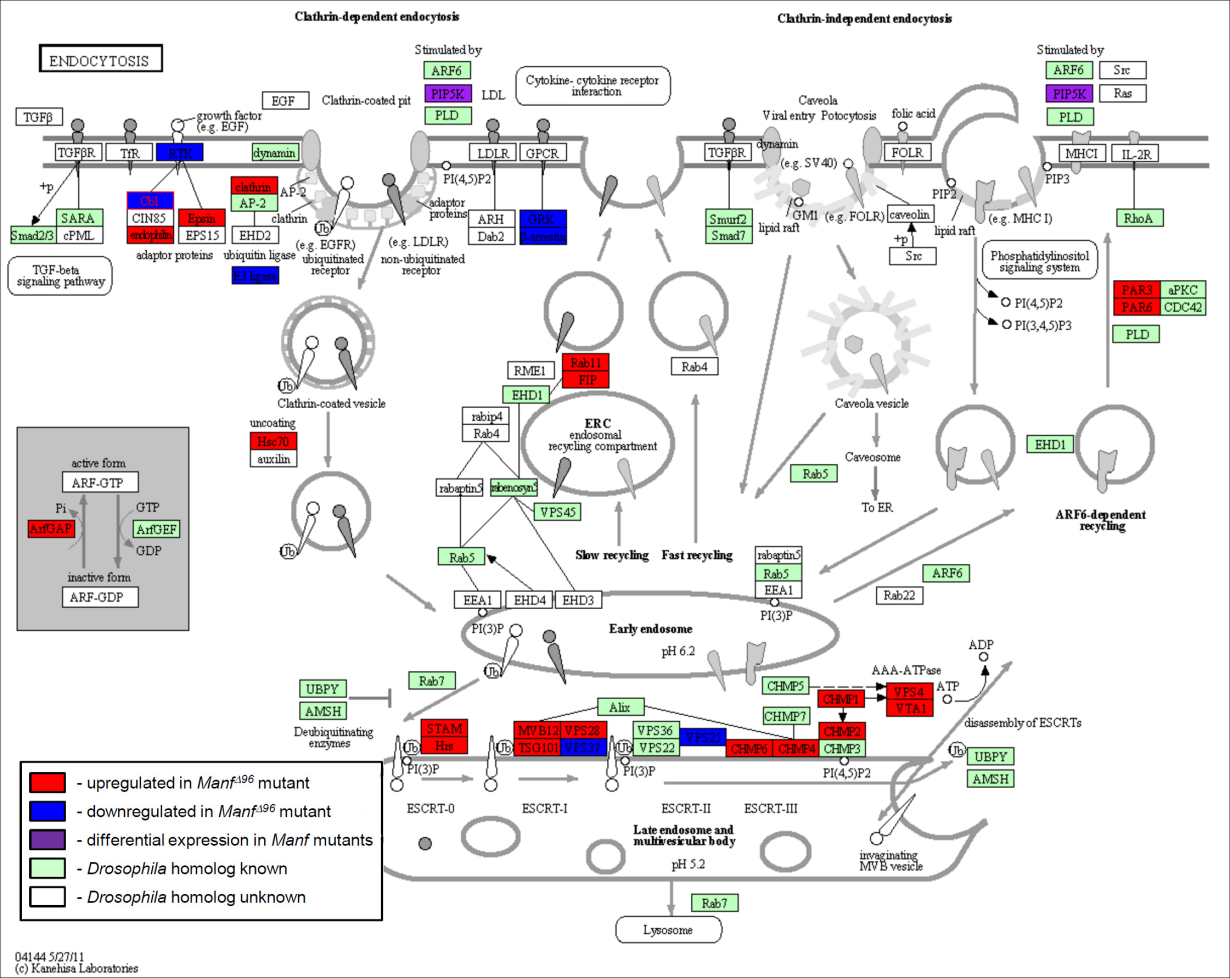
**Changes in gene expression related to endocytosis in *Manf *mutants**. An online coloured KEGG pathway showing altered gene expression in red (upregulation), blue (downregulation), or purple (altered gene expression, differences between the two mutants) boxes. The unaltered components of the pathway with *Drosophila *homologues are coloured in green. White boxes represent the pathway members identified from other organisms with an unknown *Drosophila *homologue. The complete list of altered genes is summarised in Table 9. Notice the abundant expression changes in genes encoding proteins localised close to the plasma membrane and receptor tyrosine kinase internalisation pathway components. The changes of expression were highest among genes which products localise to multivesicular body and late endosomal compartments. Two branches of recycling pathway show upregulation of gene expression of key components *PAR3*, *PAR6*, and *Rab11*.

To investigate the routes of membrane trafficking we evaluated genes involved in endocytosis. Of all *Drosophila *homologues known to be involved in endocytosis, 47% showed significant expression changes in our microarray experiment (Figure 4, Table 9). Genes coding for components of multivesicular body formation were especially altered. Several transmembrane receptors of growth factors were downregulated in *Manf *mutants and upregulated when Manf was overexpressed. PDGF- and VEGF-receptor related *Pvr *was upregulated in larvae in both lack and overexpression of Manf. *Cbl*, an E3 ubiquitin ligase and negative regulator of tyrosine kinase receptor signalling, was downregulated in mutant larvae and upregulated under Manf overexpression conditions. Two different members of endosomal recycling pathway, PAR family members and *Rab-protein 11 *(*Rab11*) were upregulated in mutants. *PAR *transcripts were upregulated by Manf overexpression as well.

**Table 9:** List of genes with altered expression according to microarray analysis related to endocytosis KEGG pathway

KEGG	Gene	embryo		larvae		
name	name	**o.ex**.	**mut**.	**o.ex**.	**mut**.	description
RTK	*htl*				**down**	Fgf receptor

RTK	*EGFR*			**up**	**down**	Egf receptor

ArfGAP	*GAP69C*		**down**	**up**	**down**	GTPase-activating protein 69C

VPS25	*VPS25*				**down**	Vacuolar protein sorting 25

beta-arrestin	*krz*			**up**	**down**	kurtz, β-arrestin

E3 ligase	*Traf6*	**up**	**down**		**down**	TNF-receptor-associated factor 6

E3 ligase	*elgi*	**up**	**down**			early girl

GRK	*GPCRK 1*	**up**	**down**			G protein-coupled receptor kinase 1

RTK	*Ret *	**up**	**down**			Ret oncogene

Cbl; E3 ligase	*Cbl*	**up**	**up**	**up**	**down**	ubiquitin mediated proteolysis

VPS37	*CG11155*	**up**	**down**	**down**		ESCRT-I complex subunit VPS37

PIP5K	*CG17471*	**up**	**down**	**down**	**up**	1-phosphatidylinositol-4-phosphate 5-kinase

CHMP4	*shrb*	**down**	**up**	**down**	**up**	shrub, charged multivesicular protein

Hsc70	*Hspc 2*		**up**	**down**	**up**	Heat shock protein cognate 2

FIP	*nuf*	**down**	**up**	**down**	**up**	nuclear fallout, Rab11 interacting protein

Epsin	*lfs*		**up**	**down**	**up**	liquid facets

endophilin	*endoB*			**down**	**up**	endophilin B

CHMP6	*Vps20*	**down**	**up**			vacuolar protein sorting 20

Clathrin	*Chc*	**down**	**up**			Clathrin heavy chain

TSG101	*TSG101*	**down**	**up**			tumor suppressor protein 101

STAM	*Stam*				**up**	signal transducing adaptor molecule

VPS28	*Vps28*		**up**		**up**	vacuolar protein sorting 28

PAR6	*par-6*		**up**	**up**	**up**	partitioning defective 6

Hsc70	*Hspc 1*			**up**	**up**	Heat shock protein cognate 1

Hsc70	*Hsp 68*	**up**	**up**			Heat shock protein 68

RTK	*Pvr*	**up**	**up**			PDGF- and VEGF-receptor related

ARFGTPase	*CG7435*	**up**	**up**			ADP ribosylation factor 84F

ArfGAP	*CG30372*	**up**	**up**			SH3 domain, ANK repeat and PH domain

ArfGAP	*cenB1A*	**up**	**up**			centaurin beta 1A

VPS4	*Vps4*	**up**	**up**			vacuolar protein sorting 4

CHMP2	*CG14542*	**up**	**up**			charged multivesicular body protein 2A

CHMP2	*CG4618*	**up**	**up**			charged multivesicular body protein 2B

MVB12	*CG7192*	**up**	**up**			ESCRT-I complex subunit MVB12

Hrs	*Hrs*	**up**	**up**			HGF regulated tyrosine kinase substrate

PAR3	*baz *	**up**	**up**			bazooka, partitioning defective 3

VTA1	*CG7967*	**up**	**up**			vacuolar protein sorting-associated protein

CHMP5	*CG6259 *		**up**			charged multivesicular body protein 5

CHMP1	*CG4108*		**up**			charged multivesicular body protein 1

ArfGAP	*cenG1A*		**up**			centaurin gamma 1A

Rab11	*Rab 11*		**up**			Rab-protein 11

Significant alterations in gene expression from wild type are shown by word code; "up" represents upregulation and "down" downregulation of gene expression. Gene name stands for the particular homologue gene name in *Drosophila*. Notice that the same KEGG identifier can lead to several different genes in *Drosophila*. The according coloured scheme for endocytosis KEGG pathway is presented in Figure 4.

To visualise Manf expression at subcellular level we used 3^rd ^instar larval salivary gland cells that are the largest ones found in *Drosophila*. In the basal part, there was partial colocalisation of Manf expression with GFP-Rab11 (Figure 1Q-S) as well as with early endosomal marker GFP-Rab5 (Figure 1N-P). In larval fat body large cells with high secretory capacity GFP-clathrin light chain (Clc), a marker for clathrin coated vesicles, colocalised with Manf in some structures (Figure 1H-J). Manf localised close to GFP-Rab7, an important player in trafficking between the early and late endosomes and lysosomes, showing weak colocalisation (Figure 1L-N). Thus Manf localises to the endosomal structures with markers Clc, Rab5, Rab7, and Rab11; but probably does not share the same protein complexes with them.

Ultrastructural analysis of *Manf^mzΔ96 ^*mutant stage 17 embryos revealed that the cells of secretory tissues such as gastric caeca, contain huge vesicles filled with cellular debris resembling multivesicular bodies and autophagosomes (Figure 3I). These structures were clearly missing in wild type embryos of the same age (Figure 3H). It is possible that these vesicles contain the misfolded proteins to be degraded or, alternatively, that the autophagy pathway is activated. The accumulation of vesicles full of debris to be degraded could be also due to the blockage in endosomal trafficking or lysosomal degradation.

### Lysosomal genes are downregulated in *Manf *mutants

Because we detected in secretory cells the accumulation of multivesicular body like structures, is it possible that the lysosomal digestion mechanism was altered. Our microarray analysis revealed transcriptional change in 45% of lysosome related genes present in the KEGG database. Many of them were downregulated in *Manf^mzΔ96 ^*embryos and some in *Manf^Δ96 ^*larvae (Table 10; Additional file 9). The ATPase V-type H^+ ^transporting subunit that maintains acidic environment in lysosomes showed downregulation in both mutants but was upregulated in Manf overexpressing larvae. The expression of other lysosomal membrane proteins and several lysosomal hydrolases was also altered.

**Table 10:** List of genes with altered expression according to microarray analysis related to KEGG lysosome pathway

KEGG	Gene	embryo		larvae		
name	name	**o.ex**.	**mut**.	**o.ex**.	**mut**.	Description
AGA	*CG10474*		**down**		**down**	N4-(beta-N-acetylglucosaminyl)-L-asparaginase

SGSH	*CG14291*			**up**	**down**	N-sulfoglucosamine sulfohydrolase

AP-3	*cm*			**up**	**down**	carmine

ARS	*CG7402*		**down**	**down**	**down**	arylsulfatase B

ATPeV	*CG7678*		**down**	**up**	**down**	V-type H+-transporting ATPase subunit I

ATPeV	*Vha100-1*	**up**	**down**			Vha100-1

ATPeV	*VhaSFD*		**down**			Vacuolar H[+]-ATPase SFD subunit

cystinosin	*CG17119*	**up**	**down**			cystinosin

GUSB	*CG2135*	**up**	**down**			beta-glucuronidase

cathepsin	*cathD*		**down**			cathepsin D, pepsin A

LAMAN	*CG6206*	**up**	**down**			lysosomal alpha-mannosidase

CLN3	*cln3*		**down**			cln3

GLA	*CG7997*		**down**			alpha-galactosidase

ATPeV	*Vha16*			**down**	**up**	Vacuolar H[+] ATPase 16kD subunit

LYPLA3	*CG31683*		**down**	**down**	**up**	lysophospholipase III

CLN1	*Ppt2*	**up**	**down**	**down**	**up**	Palmitoyl-protein thioesterase 2

LAMAN	*CG5322*	**up**	**down**	**down**	**up**	lysosomal alpha-mannosidase

LYMP	*trpml*		**down**	**down**	**up**	control of membrane trafficking of proteins and lipids

NAGA	*CG5731*	**up**	**down**	**up**	**up**	alpha-N-acetylgalactosaminidase

LIMP	*Tsp39D*			**down**	**up**	Tetraspanin 39D

LIMP	*Tsp29Fa*			**down**	**up**	Tetraspanin 29Fa

HGSNAT	*CG6903*			**down**	**up**	heparan-alpha-glucosaminide N-acetyltransferase

saposin	*Sap-r*			**down**	**up**	Saposin-related

cathepsin	*cathF*			**down**	**up**	cathepsin F

LAMAN	*CG9466*			**down**	**up**	lysosomal alpha-mannosidase

LAMAN	*CG9463*			**down**	**up**	lysosomal alpha-mannosidase

AP-3	*g*	**down**	**up**			garnet, adaptor-related protein complex 3

clathrin	*Chc*	**down**	**up**			Clathrin heavy chain

GLB	*CG9092*	**up**	**up**	**up**	**up**	beta galactosidase

GTPase	*Gie*				**up**	N-terminally acetylated Arf-like GTPase

LAMAN	*CG9465*				**up**	lysosomal alpha-mannosidase

GBA	*CG31148*	**up**	**up**		**up**	glucosylceramidase

cathepsin	*cathL *	**up**	**up**			cathepsin L

GNS	*CG18278*	**up**	**up**			N-acetylglucosamine-6-sulfatase

GGA	*Gga*	**up**	**up**			Gga

AP-1	*AP-1s*	**up**	**up**			AP-1sigma

cathepsin	*Cp1*	**up**	**up**			Cysteine proteinase-1

LYMP	*spin*	**up**	**up**			spinster, lysosomal turnover regulator

GNS	*CG30059*			**up**		N-acetylglucosamine-6-sulfatase

Significant alterations in gene expression from wild type are shown by word code; "up" represents upregulation and "down" downregulation of gene expression. ID stands for the particular homologue gene name in *Drosophila*. Notice that the same KEGG identifier can lead to several different genes. The according coloured scheme for lysosome KEGG pathway is presented in Additional File 9.

At the subcellular level, Manf colocalises partially with ER-targeted marker and very poorly if not at all with the lysosomal compartment (Figure 1F-H). Nonetheless, it is possible that the lack of Manf modifies the fusion of lysosomes with multivesicular body-like structures by some still unidentified mechanism.

### Paternal rescue of the *Manf^mzΔ96 ^*mutant embryos leads to reduction in the amount of differentially expressed genes

In *Drosophila*, substantial bulk of transcribed mRNAs and translated proteins necessary for early embryonic patterning and development are maternally contributed to the oocyte at high levels. These mRNAs and proteins are stored and used gradually during the embryogenesis and in some cases through the whole larval development. When the embryonic lack of maternal *Manf *was rescued by paternal wild type gene expression, the transcriptome changes were evident but restricted to a smaller number of genes than changes caused by the complete lack of Manf. As many as 98 genes were significantly upregulated by paternal rescue resulting in 18 functionally enriched GO term clusters (Additional file 10). We obtained GO terms related to response to stimulus and neurological process (8 genes), consisting of genes like transcription factor *pros*, *pumilio *(*pum*; encoding a mRNA binding protein involved in nervous system development), *pastrel *(*pst*; with unknown molecular function involved in memory and learning), *Rop*, *rl*, and *small optic lobes *(*sol*; calpain family peptidase). Transcripts of several genes coding membrane proteins also showed enrichment. Among them, we observed many genes coding ion-binding proteins (19%) such as *klumpfuss *(*klu*) and *odd skipped *(*odd*; DNA binding Zn-finger proteins important for embryonic nervous system development). Other enriched GO term clusters were membrane (7 genes) together with cell division, cell cycle and cytoplasm (9 genes).

Only 34 genes were downregulated by paternal rescue giving 4 functional GO term clusters (Additional file 11).

### Ubiquitous overexpression of Manf results in transcriptional activation and upregulation of genes involved in cell cycle and cell death

We used enhancer trap line *69B*-GAL4 to overexpress Manf, which we obtained as a commonly known GAL4 line with an epidermal expression pattern. After careful mapping the expression pattern of *69B*-GAL4, we detected broad GAL4 expression in other tissues than epidermis - in central nervous system (non-glial), imaginal discs (wing and eye-antennal disc), garland cells, ring gland, but neither in fat body nor in gastric caeca. Ectopic expression of Manf under *69B*-GAL4 rescues completely *Manf^Δ96 ^*mutant lethality and the rescued adults are viable and fertile if maintained as a stock [5].

When comparing the gene expression profiles between Manf overexpressing and wild type larvae we found 614 genes upregulated that could be grouped in 102 functional GO term clusters (Additional file 12). This gene set showed enrichment in processes related to regulation of gene expression, protein localisation and transport, and cell cycle (*e.g. kokopelli*, an uncharacterized cyclin involved in stem cell maintenance and *Retinoblastoma-family protein*, the human *Rb *homolog). Genes involved in regulation of cell death were also upregulated (*e.g. CG7188*, a putative Bax inhibitor, *rl*, and *klu*). According to the previous study in HeLa cells, knockdown of MANF increased cell proliferation and susceptibility to ER stress induced cell death [7]. Our results support the involvement of Manf in regulation of cell cycle and cell death offering several candidate genes for further studies.

Manf overexpression in larvae caused downregulation of 340 genes annotated in 78 functional clusters (Additional file 13). The most prominent group consisted of GO terms such as membrane, plasma membrane, signal peptide, glycoprotein, disulfide bond, glycosylation site: N-linked (GlcNAc), integral to membrane, and transmembrane (77 genes). The majority of processes related to these GO terms take place in the ER such as cleavage of the signal peptide and disulfide bond formation. The main arthropod cuticular component chitin is composed of polymerised GlcNAc residues. Another prominent group was ion binding and metal binding (70 genes). Axon guidance, cell projection organization, neuron development, axonal defasciculation, cell motion, cell recognition (20 genes) were also enriched in line with our previous results implicating the role of Manf in neuritogenesis [5].

When comparing the upregulated genes in both paternally rescued embryos and in Manf overexpressing larvae, the common represented GO term clusters were ion binding (14 genes), membrane fraction (7 genes), oxidation reduction (8 genes) and cell cycle (5 genes) (Additional file 14). All together there were 57 annotated genes commonly upregulated by Manf, among these well known genes like *Cbl, diaphanous *(*dia*, formin, essential for actin-mediated events involving membrane invagination)*, Kinesin-like protein at 68D *(*Klp68D*), *rl*, and *Rop*. Among the downregulated genes in both paternal rescue and Manf overexpression, there were only 6 genes in common *e.g. CG34384*, a diacylglycerol kinase involved in phosphoinositol signalling and glycerolipid metabolism. Conclusively, in Manf overexpression the typical growth factor signalling mediators *rl *and *Cbl *were upregulated. The links upstream of these mediators and downstream of secreted Manf still remain missing. The second cluster of genes was directly linked to membrane modifications and transport. Interestingly, the intracellular protein modification processes in ER were also enhanced by Manf overexpression. So presumably Manf has a dual role - one intracellularily in the ER, and the other extracellularly after being secreted.

### Differentially regulated genes in larval *Manf^Δ96 ^*mutant in comparison to Manf overexpression

Next, we searched for genes showing downregulation in *Manf^Δ96 ^*larval mutant and were upregulated in Manf overexpression condition, and *vice versa*. Altogether 89 probes on the microarray showed this opposite regulation resulting in 62 annotated *Drosophila *genes (Additional file 15). In this group, there were genes responsible for locomotory behaviour (3 genes), genes involved in oxidation reduction and metal binding (10 genes), in cell division (4 genes), genes coding membrane proteins (7 genes), and genes involved in insulin signalling (2 genes). Because of the diversity of GO terms and low number of genes the functional annotation clustering did not give statistically significant results.

### UPR and Parkinson's disease

**Figure 5:**
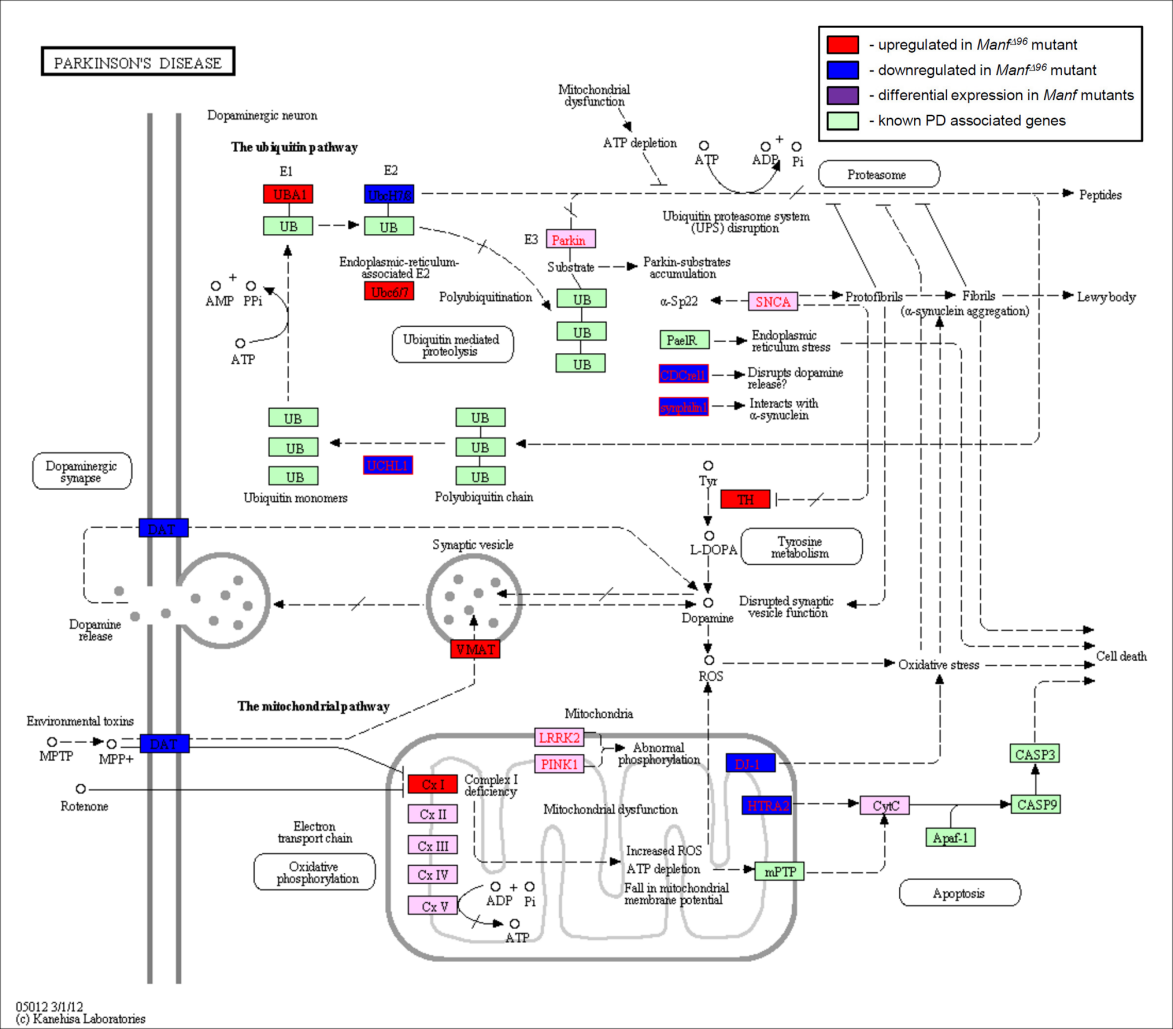
**Gene expression alterations in Manf mutants with a linkage to Parkinson's disease**. A human disease KEGG pathway coloured with *Drosophila *homologues showing altered gene expression in *Manf *mutants - filled boxes with red represent genes that are upregulated, with blue downregulated expression or with purple differently altered gene expression between two *Manf *mutants. The unaltered components in the pathway are coloured green. Note that α-synuclein (SNCA) has no homologue in *Drosophila*. The complete list of altered genes is summarised in Table 11 with some additional genes not present in KEGG pathway but have been associated with PD elsewhere. The expression of genes with a role in dopamine uptake, intracellular transport, and synthesis is altered. Mitochondrial oxidative pathway complex I encoding genes show upregulation. The other mitochondrial proteins encoded by nuclear genes involved in PD such as *Htra2 *and *DJ-1 *expression is downregulated in *Manf *mutants. Some members of ubiquitin pathway are altered as well.

Among the known genes involved in PD, 32 are conserved between mammals and *Drosophila*, and 44% of these were differentially expressed in our microarray assay (Figure 5, Table 11). Importantly, several genes from dopamine uptake (*Dopamine transporter*, *DAT*) and synthesis (*ple *coding for TH, *Ddc*, and *punch*) were differentially expressed (Table 2, Additional File 1). The expression of many genes involved in mitochondria and ubiquitin proteasome pathways were also altered. Increasing evidence indicates that organelle stress is a key event in neurodegeneration [14]. UPR is an adaptive process aiming to restore the cell homeostasis under ER stress conditions and to re-establish the properly folded protein synthesis. Under irreversible ER damage UPR initiates cell death pathway to eliminate damaged cells. Manf could be responsible for the recovery and survival pathway in UPR. In this scenario, the lack of Manf especially in the secretory cells with high rates of protein synthesis, including neurons with intensive neurotransmission, might result in shift from ER stress to UPR towards cell death. However, the mechanism how Manf dispatches this function is still unclear. Recently the structural homology between the Manf C-terminal folding and SAP domain of Ku70 has been demonstrated [34]. SAP domain of Ku70 is known to inhibit Bax-induced apoptosis [35] and *in vitro *experiments with mammalian cultured neurons showed that Manf rescues the NGF-deprivation induced cell death as efficiently as Ku70 itself [34]. On the other hand we showed that the overexpression of Manf resulted in upregulation of genes involved in oxidation reduction. The DA neurons are known to be very sensitive to oxidative stress because dopamine metabolites are highly oxidative compounds [36]. Therefore the upregulation of genes responsible for oxidation reduction could be already protective for DA neurons. Besides reactive dopamine metabolites, the mitochondrial dysfunction has been implicated in the neurodegeneration occurring in PD. We found that, in *Manf^mzΔ96 ^*mutants with degenerating DA neurites, the nuclear genes coding for mitochondrial proteins were significantly enriched among the downregulated genes.

**Table 11:** List of genes with altered expression according to microarray analysis related to KEGG pathway of Parkinson's disease

KEGG	Human	embryo		larvae		Flybase	
name	gene	**o.ex**.	**mut**.	**o.ex**.	**mut**.	ID	Description
TH	*TH *		**up**			*CG10118*	pale, tyrosine hydroxylase

VMAT	*VMAT*				**up**	*CG6139*	vesicular monoamine transporter

DAT	*SCL6A3 *	**up**	**down**			*CG8380*	dopamine transporter

HTRA2	*HTRA2 *	**up**	**down**			*CG8464*	HtrA serine peptidase 2

NDUFV1	*NDUFV1 *	**up**	**down**			*CG9140*	NADH dehydrogenase

SNCAIP	*SNCAIP *			**up**	**down**	*CG5424*	forked, actin binding α synuclein interacting

CDCREL1	*PNUTL1 *			**up**	**down**	*CG7238*	septin interacting protein 1

CytC	*CYCS *			**up**	**down**	*CG14235*	cytochrome-c oxidase

UQCRH	*UQCRH *	**up**		**up**	**down**	*CG30354*	ubiquinol-cytochrome-c reductase

	*SIP2 *			**up**	**down**	*CG9188*	septin interacting protein 2

	*SLC25A6 *			**up**	**down**	*CG1683*	mitochondrial adenine nucleotide translocase 2

Uchl1	*UCHL *			**up**		*CG4265*	Ubiquitin carboxy-terminal hydrolase

mPTP	*VDAC2 *	**up**	**up**	**up**	**down**	*CG17137*	Porin2, voltage-gated anion channel

ATPase	*ATP5EP2 *			**up**		*CG31477*	mitochondrial ATPase ε subunit, F1 complex

NDUFA2	*NDUFA2 *			**up**	**up**	*CG15434*	NADH dehydrogenase

	*SEP4 *		**up**		**up**	*CG9699*	Septin 4

Ube2J2	*UBE2J2 *			**down**	**up**	*CG5823*	ubiquitin-protein ligase

NDUFV2	*NDUFV2 *				**up**	*CG5707*	NADH dehydrogenase

	*UBE2H *				**up**	*CG2257*	Ubc-E2H, ubiquitin-protein ligase

Ube2L3	*UBE2L3 *			**down**	**down**	*CG12799*	Ubiquitin conjugating enzyme 84D

	*UBQLN1 *			**down**		*CG14224*	EF1B ubiquitin-assoc. translation elongation factor

Ubh4	*UCHL *				**down**	*CG3431*	Ubiquitin C-terminal hydrolase

	*USP36 *				**down**	*CG5505*	scrawny, ubiquitin protease

	*SIP3 *				**down**	*CG1937*	septin interacting protein 3

Uba1	*UBA1 *		**up**			*CG1782*	ubiquitin activating enzyme 1

CytC	*CYCS *	**up**	**up**			*CG2249*	cytochrome-c oxidase

	*SEP1 *		**up**			*CG1403*	Septin-1

	*UBE2E2 *		**up**			*CG6720*	Ubiquitin conjugating enzyme 2

DJ-1	*DJ-1 *		**down**			*CG6646*	DJ-1α

CytC	*CYCS *		**down**			*CG13263*	cytochrome c distal

CytC	*CYCS *		**down**			*CG14028*	cyclope, cytochrome-c oxidase

CytC	*CYC1 *		**down**			*CG4769*	electron transporter

ATPase	*ATP5G2 *	**down**	**down**			*CG1746*	hydrogen-exporting ATPase

ATPase	*ATP5E *		**down**			*CG9032*	stunted, hydrogen-exporting ATPase

Significant alterations in gene expression from wild type are shown by word code; "up" represents upregulation and "down" downregulation of gene expression. Notice that the same KEGG identifier can lead to several different genes. Several *Drosophila *genes associated with processes in PD missing from KEGG pathway were added. The according coloured scheme for Parkinson's disease KEGG pathway is presented in Figure 5.

## Conclusions

We have studied the gene expression changes of two slightly different *Manf *mutants in *Drosophila*. Surprisingly, the expression profiles of embryonic lethal *Manf^mzΔ96 ^*and larval lethal *Manf^Δ96 ^*mutants were quite diverse. It might be due to the dissimilar roles of *Manf *gene during embryonic and larval stages or it indicates the difference between the absolute lack of Manf versus gradual diminishing of maternally contributed stores of *Manf *gene products. Our microarray analysis followed by functional annotation clustering revealed statistically significant enrichment related to metabolism and membrane transport and transporters. The observed changes in membrane traffic were supported by ultrastructural studies of *Manf^mzΔ96 ^*mutant. More than 40% of known *Drosophila *genes related to ER and UPR showed altered expression levels in *Manf *mutants. We found changed expression of several genes known to be associated with processes altered in PD such as oxidative phosphorylation, mitochondrial function, dopamine metabolism and uptake, and protein ubiquitinaton. The lack of Manf in *Manf^mzΔ96 ^*mutant embryos resulted in massive upregulation of stress, defense, and immune response related genes as well as genes involved in proteolysis and cell death. Overexpression of Manf resulted in upregulation of genes involved in oxidation reduction, an important process to protect DA neurons from oxidative stress. Thus, our results support the previously reported findings in mammalian cells that upregulation of Manf is important in UPR and could be protective for the cell. It is also evident that ER stress leads to UPR and cell death in the absence of Manf. These effects were less drastic when Manf was gradually abolished in *Manf^Δ96 ^*mutant larvae and related to several metabolic processes and downregulation of genes involved in replication, transcription and splicing. Still, stress and defense related genes were enriched among the upregulated genes of both *Manf *mutants.

## Methods

### Fly strains

*Drosophila melanogaster *adults were maintained at 25°C on malt and wholemeal flour based standard food, for egg laying apple juice agar plates with yeast paste were used. Wild type flies were *w^-^*, all genotypes used were of *w^- ^*background: *hs70Flp/+;; FRT^82B^Manf^Δ96^/Manf^Δ96^*, *Manf^Δ96^/TM3 Sb Ser twi > GFP*. For ectopic overexpression *69B-*GAL4 [37] and UAS-*DmManf^133 ^*(3^rd ^chromosome insertion) were used. As an ER marker ER-targeted *sqh*-EYFP flies were used. UAS-GFP-*rab5*, UAS-GFP-*rab11*, UAS-*chc*-GFP, and UAS-GFP-*rab7 *were obtained from C. Samakovlis and *ppl *> GAL4 from V. Hietakangas. Other fly lines were obtained from Bloomington *Drosophila *Stock Center or generated by us [5].

### RNA isolation

For RNA extraction, embryos were collected from apple juice plates, washed with embryowash, dechorionated by standard bleach treatment and washed thoroughly. Both embryos and larvae were separated and picked by phenotype and staged under microscope onto fresh apple juice plates, collected into Eppendorf tubes and snap frozen. Total RNA was extracted with Qiagene RNAeasy extraction Kit (Qiagene) according to manufacturer's recommendations, treated with RNase-free DNase (Promega) 15 min at 37°C and purified by RNA clean-up kit (Macherey-Nagel). RNA was quantified using a NanoDrop-1000 spectrophotometer and RNA quality was monitored by the Agilent 2100 Bioanalyzer (Agilent Technologies).

### Microarray experiments

For microarray experiment three biologically independent samples for each genotype were used. Diluted spike controls (Agilent) were added to 1 μg of total RNA samples and *in vitro *transcribed and labeled with Amino Allyl MessageAmp™ II aRNA Amplification Kit (Ambion/Applied Biosystems). The dyes used were cyanine 3 (Cy3), cyanine 5 (Cy5) or Alexa 488, as previously described [38]. Dye incorporation and received aaRNA yield were monitored by the NanoDrop ND-1000 Spectrophotometer.

### Hybridisation

5 μg of each differentially labelled aaRNA was fragmented at 60°C for 30 min in a reaction volume of 55 μl containing Agilent fragmentation buffer and 2x Agilent blocking agent following the manufacturer's instructions. On completion of the fragmentation reaction, 55 μl of 2x Agilent hybridization buffer was added to the fragmentation mixture and hybridized to Agilent's fruit fly Microarray Kit 4x44k, P/N G2519F (Agilent Microarray Design ID 018972) for 17 hours at 65°C in a rotating Agilent hybridization oven. After hybridization, microarrays were washed 1 min at room temperature with GE Wash Buffer 1 (Agilent) and 1 min with 37°C GE Wash buffer 2 (Agilent), then dried immediately by brief centrifugation. The slides were then scanned by Axon 4200AL scanner.

### DNA microarray analysis

The microarrays images were segmented and the median intensity of each spot was estimated by the software GenePixPro^® ^6.0 (Axon). The data were then imported into R software http://cran.r-project.org/ and preprocessed by the BioConductor package Limma [39]. Linear model followed by moderated t-test was utilized for finding the differentially expressed genes (P-value < 0.01 after Benjamini and Hochberg correction) between *Manf^Δ96^*, *Manf^mzΔ96^*, *Manf^mΔ96^/+*, *69B > Manf^133 ^*and *w^-^*. Lists of significant genes were screened by the DAVID 6.7 annotation tools [40,41] in order to find over-represented biological themes. Default DAVID parameters were used. To identify the pathways altered, the online tool available from Kanehisa laboratories, KEGG Mapper was used [42]. All microarray data are MIAME compliant and available at the NCBI GEO database (ID GSE28820).

### Quantitative PCR

For qPCR, independent biological samples were used for RNA extraction. 4 μg of total RNA was reverse transcribed with MMLV reverse transcriptase (Promega) according to manufacturer's instructions using oligo(dT) primers. The primers were designed with the help of Universal ProbeLibrary Assay Design System (Roche Applied Science) and are listed in Additional file 16. qPCR was performed with Lightcycler 480 real-time PCR system (Roche Diagnostics) with the help of pipeting robot Robotics4 (Corbett Robotics) on 384-well plates using Lightcycler 480 SYBR Green I Master complemented with 5 pmol of primers and cDNA corresponding to 40 ng of total RNA used in reverse transcription. Three replicates for each reaction were included in the PCR runs. Results were analysed with Lightcycler software version 1.5.0.39.

### Transmission electron microscopy and immunohistochemistry

The embryos for TEM were treated as previously described [43]. The whole larvae were subjected to high pressure freezing to visualise the cuticle layers as described earlier [44]. The primary antibodies used were rabbit phospho-eIF2α (Ser51) antibody (1:1000, Cell Signaling Technology), mouse α-DmTubulin (1:1000, Sigma) and rabbit α-DmManf (1:1000) [5]. Immunohistochemistry and imaging were performed as previously described [5]. To visualise the lysosomes, Lysotracker Red DND99 (Invitrogen) was used. The red colour of Alexa568 dye was changed to magenta in order to help colour blind people to distinguish it in the combinations with green.

### Western blotting

For Western blotting about 100 embryos of stage 17 were collected, genotyped, and homogenised in 10 mM HEPES, 1 mM EDTA, 0.25 M sucrose homogenising buffer, pH = 7.3 in the presence of protease inhibitor cocktail (Complete Mini, Roche). The concentration of proteins was measured with Bio Rad protein assay D_C _reagents. The equal amounts of total protein were mixed with 3 × Laemmli loading buffer and boiled at 99°C for five minutes. Up to 6 μg of total protein were loaded per lane to SDS-acrylamide gel. Western blotting was further proceeded according to the standard manufacturer's instructions (Amersham Biosciences). For the quantification of Western blotting results ImageJ analysis software was used. Quantification was based on area measurements and intensity calculations in comparison with the anti-tubulin loading control.

## Competing interests

The authors declare that they have no competing interests.

## Authors' contributions

MP, TIH, DG, and PA designed the research, MP performed the laboratory experiments, DG scanned the slides and made the analysis and comparisons of the raw scanned microarray data, MP with the help of RL completed the annotation, and MP made functional annotation clustering analysis, all other laboratory experiments and wrote the draft manuscript. All authors read, made corrections and approved the manuscript.

## Supplementary Material

Additional file 1 **Table of all obtained qPCR results**. The Excel file contains two separate sheets, one for the data obtained from stage 17 embryos and the other for the data obtained from 29-50 hr AEL larvae. Click here for file

Additional file 2 **DAVID functional annotation clustering analysis of downregulated genes in embryonic *Manf^mzΔ96 ^*mutants**. An Excel file; 1191 genes were downregulated and grouped into 105 functional clusters. Click here for file

Additional file 3 **DAVID functional annotation clustering analysis of upregulated genes in embryonic *Manf^mzΔ96 ^*mutants**. An Excel file; 1243 genes were upregulated and grouped into 230 functional clusters. Click here for file

Additional file 4 **DAVID functional annotation clustering analysis of downregulated genes in larval *Manf^Δ96 ^*mutants**. An Excel file; 690 genes were downregulated and grouped into 140 functional clusters. Click here for file

Additional file 5 **DAVID functional annotation clustering analysis of upregulated genes in larval *Manf^Δ96 ^*mutants**. An Excel file; 682 genes were upregulated and grouped into 122 functional clusters. Click here for file

Additional file 6 **DAVID functional annotation clustering analysis of common downregulated genes in *Manf^mzΔ96 ^*and *Manf^Δ96 ^*mutants**. An Excel file; 208 genes were downregulated in both *Manf *mutants and grouped into 47 functional clusters. Click here for file

Additional file 7 **DAVID functional annotation clustering analysis of common upregulated genes in *Manf^mzΔ96 ^*and *Manf^Δ96 ^*mutants**. An Excel file; 229 genes were upregulated in both *Manf *mutants and grouped into 45 functional clusters. Click here for file

Additional file 8 **Exocytosis and SNARE complex is altered in *Manf *mutants**. A pdf file; an online coloured KEGG pathway showing altered gene expression in either red (upregulation), blue (downregulation), or in purple (altered gene expression) boxes. The unaltered known *Drosophila *homologues to identified components from other organisms are presented in green-filled boxes. The complete list of altered genes is summarised in Table 8. Click here for file

Additional file 9 **Lysosomal degradation is altered in *Manf *mutants**. A pdf file; an online coloured KEGG pathway showing altered gene expression in either red (upregulation), blue (downregulation), or in purple (altered gene expression) boxes. The unaltered known *Drosophila *homologues to identified components from other organisms are presented in green-filled boxes. The complete list of altered genes is summarised in Table 10. Click here for file

Additional file 10 **DAVID functional annotation clustering analysis of upregulated genes in *Manf^mzΔ96 ^*mutant paternal rescue**. An Excel file; 98 genes were upregulated when maternal lack of *Manf *gene products was rescued paternally and grouped into 18 functional clusters. Click here for file

Additional file 11 **DAVID functional annotation clustering analysis of downregulated genes in *Manf^mzΔ96 ^*mutant paternal rescue**. An Excel file; only 34 genes were downregulated when maternal lack of *Manf *gene products was rescued paternally and grouped into 4 functional clusters. Click here for file

Additional file 12 **DAVID functional annotation clustering analysis of upregulated genes in Manf ectopic overexpression by *69B*-GAL4**. An Excel file; 614 genes were upregulated and grouped into 102 functional clusters. Click here for file

Additional file 13 **DAVID functional annotation clustering analysis of downregulated genes in Manf ectopic overexpression by *69B*-GAL4**. An Excel file; 340 genes were downregulated and grouped into 78 functional clusters. Click here for file

Additional file 14 **DAVID functional annotation clustering analysis of common upregulated genes in *Manf^mzΔ96 ^*paternally rescued embryos and in Manf ectopically overexpressing larvae**. An Excel file; 57 genes were upregulated in both *Manf *mutants and grouped into 9 functional clusters, 14 GO terms remained unclustered. Click here for file

Additional file 15 **Complete list and DAVID functional annotation clustering analysis of genes downregulated in *Manf^Δ96 ^*mutants and upregulated in Manf ectopic overexpression and *vice versa***. An Excel file; 46 genes were downregulated in larval *Manf^Δ96 ^*mutants and upregulated in Manf ectopic overexpression, *vice versa *16 genes were upregulated in *Manf^Δ96 ^*mutants and downregulated in Manf ectopic overexpression. In the first sheet complete list of genes together with fold change logarithmic (FClog) results is presented. In the second sheet results of DAVID functional annotation clustering analysis with 9 clusters is shown. Click here for file

Additional file 16 **List of all designed primers used for qPCR**. Click here for file
