# Supplementary figures and images for: Gene expression analysis of Drosophilaa Manf mutants reveals perturbations in membrane traffic and major metabolic changes
Source: BMC Genomics. 2012 Apr 11;13:134. doi: 10.1186/1471-2164-13-134 (PMC3364883; doi:10.1186/1471-2164-13-134)

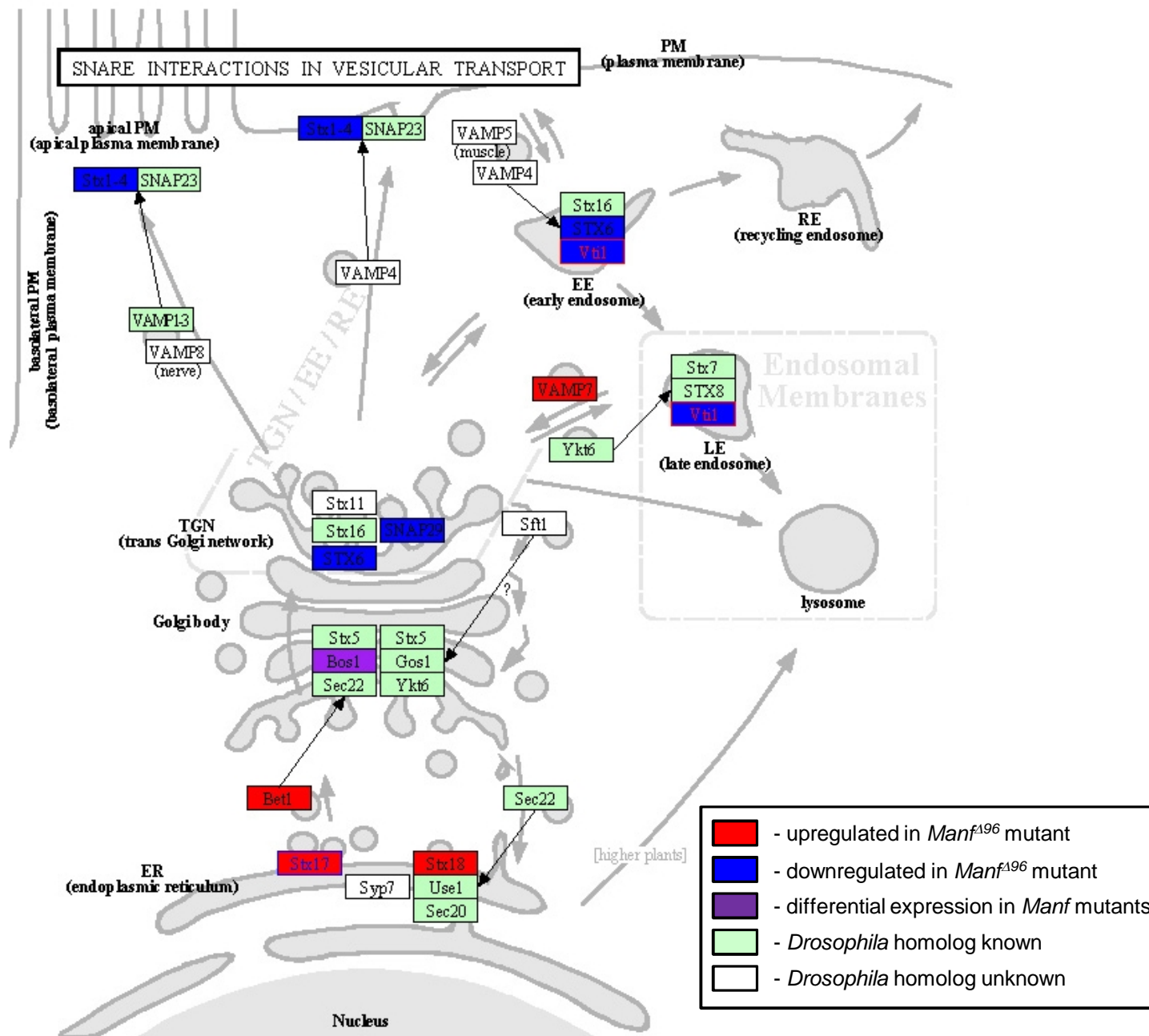

04130 3/4/10  
(c) Kanehisa Laboratories

Additional File 8.

Supplement: Additional file 8 — Exocytosis and SNARE complex is altered in Manf mutants. A pdf file; an online coloured KEGG pathway showing altered gene expression in either red (upregulation), blue (downregulation), or in purple (altered gene expression) boxes. The unaltered known Drosophila homologues to identified components from other organisms are presented in green-filled boxes. The complete list of altered genes is summarised in Table 8. [file 1471-2164-13-134-S8.PDF]
