# Supplementary material for: Gene expression analysis of Drosophilaa Manf mutants reveals perturbations in membrane traffic and major metabolic changes
Source: BMC Genomics. 2012 Apr 11;13:134. doi: 10.1186/1471-2164-13-134 (PMC3364883; doi:10.1186/1471-2164-13-134)
Supplement: Additional file 9 — Lysosomal degradation is altered in Manf mutants. A pdf file; an online coloured KEGG pathway showing altered gene expression in either red (upregulation), blue (downregulation), or in purple (altered gene expression) boxes. The unaltered known Drosophila homologues to identified components from other organisms are presented in green-filled boxes. The complete list of altered genes is summarised in Table 10. [file 1471-2164-13-134-S9.PDF]

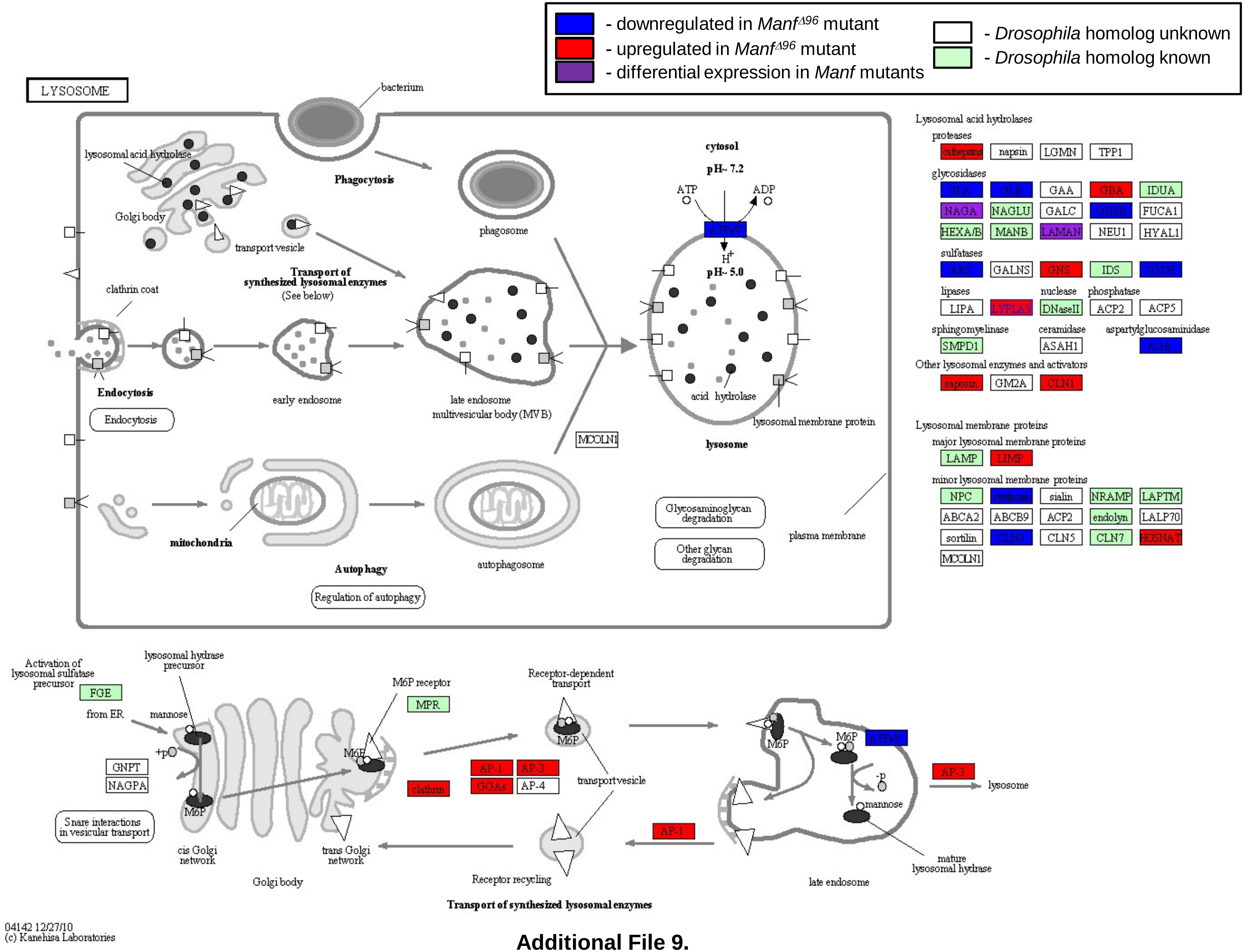

Additional File 9.
