# Supplementary material for: Gene expression analysis of Drosophilaa Manf mutants reveals perturbations in membrane traffic and major metabolic changes
Source: BMC Genomics. 2012 Apr 11;13:134. doi: 10.1186/1471-2164-13-134 (PMC3364883; doi:10.1186/1471-2164-13-134)
Supplement: Additional file 16 — List of all designed primers used for qPCR. [file 1471-2164-13-134-S16.PDF]

Gene name	ID	left	right
CG5810	CG5810	ccaaagctgcatgagttgaa	ggccaagtaaagatgctcca
cycle	Cyc	gcgatggaagttcaggagtt	tgtgattttgcttgcgattt
insulin-like peptide 5	Ilp5	gccttgatggacatgctga	cataatcgaataggcccaagg
Insulin-like receptor	InR	acgacaacaaaaccgttgc	ttcacgtgatctcaatcatgc
no optic lobe	Nol	gctactgatccagccacga	ggcgaagtggaagtggtg
Sphingosine kinase 2	Spk2	cagctacaaaggccgagtgt	tccactggtggttccttctt
Ect3	Ect3	tgcgtgtagctgcaatcg	cagctcctagcagcggtagt
Cadherin 99C	Cad99C	ggggcagacgaacatcat	gtcggctgtccgatgaaa
diaphanous	Dia	gattatgtggcacttgaaaggtaa	ccattctgcaggtattccaca
sulfateless	Slf	aagctgtcgatttgagtagcaa	gactgtccactcgcaatcag
Ras-related protein	Rala	aggcaacccaggaattcag	tgcgcttatcattcagatcg
CG1607	CG1607	agccgtctgttgcatcctt	catatgtaaagatatcctgcacagc
Heat shock protein cognate 2	Hspc2	agacggagcgcttgattg	tcgcttggcatcgaaaac
Ras opposite	ROP	aagctgatgaacgaggtggt	acgtaacgccctgcttttt
klumpfuss	Klf	ctcggccaccgtctaaatc	tgggattcttcctcgctatc
pathetic	Path	atgtgctccggtctcatcat	cttgtgtccgcattttacca
CG14879	CG14879	gcctgatgtgaagttctttgc	tgacaaagttcaccgagaattg
Pak3	Pak3	aaatttgttttgtgtggacgaa	tcctcgctgactcgtgct
Rab escort protein	Rep	agcagtttgacctggtcgtc	accgatttgcctatcctgct
pipe	Pip	cctcaaggagatggaggtga	ggtgttgatgtcatcgtgct
DnaJ homolog	CG9828	gcagtcgggactccacttat	gcgtcaaactgttgatgtcc
Heat shock protein 83	CG1242	tctgtgaatagaacgaaaaacataca	tgatgatcagggacatcagc
ubisnap	CG11173	ctctggactctaccaacaaaagc	tgtgatgtcttctccagttgct
CG6000A	CG6000	gcaatctcctccaacgttttt	acgtcgtagtccacgattcc
CG6000B	CG6000	ttcatttgttgttctctgtgatttta	
CG10420	CG10420	cagacgattgcggaaggt	tgactctgcagcgacgtg
Cbl	CG7037	tttggaagagcaacttcggta	gccggagattatgggatgta
sip3	CG1937	ggcaagctgctaagcaaga	tccagcagatgctcaaactc
Su(var)2-10	CG8068	ggactacacgcgaggcttaat	agcatagtggtggctatttcg
Ubc-E2H -A	CG2257	cacacaccgagagaaatcaaaat	gcattcgtcaactctcactgg
Ubc-E2H- B	CG2257	acaaagtcacaaaaatccgtctt	
Ubc-E2H- C	CG2257	gagggccagcaacaactaaa	agagcaaaacgggagctg
Ubiquilin	CG14224	cgtgatgccaacttgcag	tggcttagccttgttaactcaa
Uchl	CG4265	tcccgtaaaggcgttcatt	gcgcggtgcttctcatag
Hrs	CG2903	gctaagtccggaccacgtc	cttgcgagctggtgtctg
lqf	CG8532	actggatgacccatggaaag	aactcgggggattgtttctta
Atg8a	CG32672	ttcattgcaatcatgaagttcc	gggagccttctcgacgat
PEK	CG2087	ggctcgaacactgcgtaga	ccatcaagcgtggaaatgta
Rab-2	CG3269	caaatacatcattatcggagacaca	atcgcttgtccgtgaactg
Syx1A	CG31136	gaccaacgacgatgagctg	tgcgtgaacacagacgagtt
Pi3K92E	CG4141	tgtggaaacgcgatggta	atgttggcaatggtttctgc
cul-1 (lin-19)	CG1877	gcaactgcaggacaacacac	ttcgccttgagcagtatttg
CG9188	CG9188	ggaatctttaattatcaatggaatcg	ggcaaaggagttcgttcatc

Additional File 16.
